# Variations of chromosomes 2 and 3 gene expression profiles among pulmonary telocytes, pneumocytes, airway cells, mesenchymal stem cells and lymphocytes

**DOI:** 10.1111/jcmm.12429

**Published:** 2014-10-02

**Authors:** Minghuan Zheng, Xiaoru Sun, Miaomiao Zhang, Mengjia Qian, Yonghua Zheng, Meiyi Li, Sanda M Cretoiu, Chengshui Chen, Luonan Chen, Dragos Cretoiu, Laurentiu M Popescu, Hao Fang, Xiangdong Wang

**Affiliations:** a Biomedical Research Center, Minhang Hospital & Zhongshan Hospital, Fudan University Center for Clinical BioinformaticsShanghai, China; b Department of Pulmonary Medicine, The First affiliated Hospital, Wenzhou Medical UniversityWenzhou, China; c State Key Lab of Systems Biology, Chinese Academy of ScienceShanghai, China; d Department of Cell Biology and Histology, Carol Davila University of Medicine and PharmacyBucharest, Romania; e Victor Babeş National Institute of PathologyBucharest, Romania; f Department of Anesthesiology, Zhongshan Hospital and Jinshan Hospital of Fudan UniversityShanghai, China

**Keywords:** TCs, mesenchymal stem cells, fibroblasts, chromosome 2, chromosome 3, lung

## Abstract

Telocytes (TCs) were identified as a distinct cellular type of the interstitial tissue and defined as cells with extremely long telopodes (Tps). Our previous data demonstrated patterns of mouse TC-specific gene profiles on chromosome 1. The present study focuses on the identification of characters and patterns of TC-specific or TC-dominated gene expression profiles in chromosome 2 and 3, the network of principle genes and potential functional association. We compared gene expression profiles of pulmonary TCs, mesenchymal stem cells, fibroblasts, alveolar type II cells, airway basal cells, proximal airway cells, CD8^+^T cells from bronchial lymph nodes (T-BL), and CD8^+^ T cells from lungs (T-LL). We identified that 26 or 80 genes of TCs in chromosome 2 and 13 or 59 genes of TCs up-or down-regulated in chromosome 3, as compared with other cells respectively. Obvious overexpression of Myl9 in chromosome 2 of TCs different from other cells, indicates that biological functions of TCs are mainly associated with tissue/organ injury and ageing, while down-expression of Pltp implies that TCs may be associated with inhibition or reduction of inflammation in the lung. Dominant overexpression of Sh3glb1, Tm4sf1 or Csf1 in chromosome 3 of TCs is mainly associated with tumour promotion in lung cancer, while most down-expression of Pde5 may be involved in the development of pulmonary fibrosis and other acute and chronic interstitial lung disease.

## Introduction

Telocytes (TCs) were first described as a distinct cell type in 2010 1,2, followed by a significant growing of research globally 3,4, as detailed in www.telocytes.com. TCs were found in multiple tissues and organs, such as heart 5–7, kidney 8 and urinary tract 9,10, skin 11,12 eye 13, mammary gland 14, digestive tract 15,16, skeletal muscles 17 and neuromuscular spindles 18, uterus 19–22 and placenta 2, liver 23 and gall bladder 24,25, pleura 26, trachea 27 and lungs 28. TCs are mainly recognized and characterized by electron microscopy, the only technique able to highlight their characteristic extensions – the telopodes (Tps), consisting of thin segments – podomers, alternating with dilated regions – podoms 1. Other characteristics of Tps include: (*i*) the unusual and varied length, between tens and thousands of micrometres; (*ii*) the branching network pattern, forming a labyrinthine system; (*iii*) the communications through homo-and heterocellular junctions exosome and ectosome release 6,20,22. TCs were found to link nerve fibres, blood vessels, secretory acini and exocrine epithelial ducts 29–31, and different cell types, *e.g*. macrophages, lymphocytes, mast cells, stem cells 32–34. TCs form 3-dimensional networks within organs/tissues 20,35. Networks integrity may be affected in many pathological conditions, such as systemic sclerosis 36, skin basal and squamous cell carcinomas 37 and Crohn's disease 38.

Telocytes differ from fibroblasts (Fbs) and mesenchymal stem cells (MSCs) as demonstrated by miR signatures and genetic profiles 15,39,40. Proteomic signatures of the TCs are also supportive for the uniqueness and helpful in understanding of the functions. The data from omics studies demonstrated that elements within TCs are involved in (*i*) intercellular signalling, (*ii*) mechanical sensing and mechanochemical conversion task, (*iii*) tissue homoeostasis and remodelling/renewal, (*iv*) anti-oxidative stress and anti-ageing cellular mechanisms, (*v*) cancer cell proliferation through the inhibition of apoptosis 40,41. Our recent work explored patterns of mouse TC-specific gene profiles on chromosome 1 and showed important roles for TCs in the prevention of tissue inflammation and fibrogenesis, development of lung inflammatory diseases or modulation of immune cell responses 42. However, dominant patterns and specificity of gene and protein profiles of TCs which are different from other cells existed in the lung is still not completed and unclear.

The present study undertakes an in-depth analysis to find out the characters and patterns of TC-specific or TC-dominated gene expression profiles in chromosome 2 and 3, investigate the network of principle genes, and explore potential functional association. Comparisons are made among pulmonary TCs, MSCs, Fbs, alveolar type II cells (ATII), airway basal cells (ABCs), proximal airway cells (PACs), CD8^+^T cells from bronchial lymph nodes (T-BL) and CD8^+^ T cells from lung (T-L), which may interact with TCs in the lung and trachea. Furthermore, we applied the most complete reference library of the National Center for Biotechnology Information (NCBI) Gene Expression Omnibus database to identify key functional genes, and characteristic networks by bioinformatics tools.

## Material and methods

### Isolation and culture

Telocytes were isolated from the lung tissues of mice, primary cultured in a concentration of 1 × 10^5^ cells/cm^2^, and harvested on days 5 (TC D5) and on days 10 (TC D10), as described previously 43. RNA isolation, preparation, labelling, and hybridization were performed for DNA microarray (The Mouse 4 × 44K Gene Expression Array, Agilent, Shanghai, China). About 39,000+ mouse genes and transcripts represented with public domain annotations were gained, according to the protocol of One-Color Microarray-Based Gene Expression Analysis. The hybridized arrays were washed, fixed and scanned by the Agilent DNA Microarray Scanner (part number G2505B).

### Data collection and mining

We selectively collected gene expression profiles of pulmonary TCs on days 5 (TC D5) and 10 (TC D10), Fbs, MSCs, from our study 43, ATII, ABCs, PACs, T-BL and T-L, from the NCBI Gene Expression Omnibus database (GSE6846 44, GSE27379 45, GSE28651 46). The microarray was composed of 45,101 probes. We eliminated the probe sets without corresponding official symbol, leaving 39,417 probes and 21,680 genes.

The gene expression profiles are from our earlier study, which are composed of 23,861 probes, of pulmonary TCs on days 5 and 10, Fbs and MSCs are composed of 23,861 probes 43. There were 13,236 probes and 11,545 genes after further eliminating the probes without corresponding official symbol, which we focused on in the present study. From the total of 11,545 genes, 917 genes of the chromosome 2 and 567 genes of the chromosome 3 were analysed.

### Identification of differentially expressed genes

There are about 20,000–25,000 genes in mouse, of which about 85% are similar with humans, and the propensity of functional changes was reflected in different levels of the gene expression in particular cell types. We used gene expression profiles between mouse lung cells to seek for the specific regulated and identify genes specific to TCs and their function. The fold change was utilized to identify differentially expressed genes or simply differential genes. Up-or down-regulated folds of TCs genes were calculated as compared with other cells and subtracted its own multiple of TCs, after the average of gene expression in each cell was obtained from the raw data of multi-databases, as shown in Data S1.

## Results

The final data analysis by bioinformatics tools showed that in chromosome 2*,* 26 genes were overexpressed in TCs, as compared with those in other cells (Table1). Among them, 20 genes (1110008F13Rik, 2310003F16Rik, 2900064A13Rik, Abl1, Ass1, Commd3, Commd7, Creb3l1, Dlgap4, Edf1, Id1, Manbal, Mocs3, Psmc3, Sdccag3, Slc39a13, Snai1, Spc25, Tubb2c, Srxn1) were overexpressed between 0 and 1 folds (Table1A). Four genes, Dbndd2 (Dysbindin domain-containing protein 2), Fbn1 (fibrillin 1), Tfpi (tissue factor pathway inhibitor) and Ak1 (adenylate kinase 1) genes, were overexpressed one-to-fourfold, in both TC D5 and TC D10, as compared with other cells (Table1B). Highest overexpressed gene, Myl9 (myosin, light chain 9), was over fourfold up-regulated in both TC D5 and TC D10 compared with other cells (Table1C). 80 genes in TCs were down-regulated, as compared with other cells (Table2). Of them, Gzf1, Pltp, Polr1b, Tasp1, Zbtb34 and Zfp120 were down-regulated more than onefold in TCs compared with other cells.

**Table 1:** Summary of genes expressed preferentially in TCs, as compared with others

Compared pairs/fold up-regulated	>0	>1	>4
TC5 *versus* others	64	18	2
TC10 *versus* others	56	13	4
TCs *versus* others	26	6	2
Gene symbol	Folds (TC5 *versus* others/TC10 *versus* others)						
	Fibroblast	Stem	ATII	CD8_T_BL	CD8_T_LL	Basal_cell	Duct_cell
(A) Genes up-regulated between 0-and 1-folds in TCs as compared with others							
1110008F13Rik	−0.41/−0.18	−0.46/−0.25	−0.73/−0.72	−0.79/−0.80	−0.85/−0.85	−0.91/−0.91	−0.91/−0.92
2310003F16Rik	−0.40/−0.04	−0.49/−0.19	−0.97/−0.97	−0.52/−0.46	−0.56/−0.50	−0.82/−0.80	−0.86/−0.84
2900064A13Rik	−0.13/−0.21	−0.41/−0.46	−0.37/−0.58	−0.29/−0.54	−0.30/−0.54	−0.24/−0.51	−0.19/−0.47
Abl1	−0.75/−0.67	−0.43/−0.23	−0.76/−0.76	−0.29/−0.32	−0.70/−0.71	−0.82/−0.83	−0.89/−0.90
Ass1	−0.44/−0.34	−0.96/−0.95	−0.82/−0.84	−0.94/−0.95	−0.80/−0.83	−0.90/−0.92	−0.80/−0.84
Commd3	−0.63/−0.13	−0.63/−0.54	−0.56/−0.62	−0.78/−0.59	−0.76/−0.60	−0.13/−0.34	−0.24/−0.65
Commd7	−0.29/−0.56	−0.62/−0.91	−0.58/−0.49	−0.53/−0.99	−0.54/−0.99	−0.24/−0.87	−0.60/−0.89
Creb3l1	−0.65/−0.66	−0.93/−0.44	−0.46/−0.35	−0.99/−0.18	−0.99/−0.52	−0.86/−0.25	−0.88/−0.29
Dlgap4	−0.79/−0.98	−0.65/−0.96	−0.44/−0.77	−0.28/−1.00	−0.58/−0.96	−0.34/−0.37	−0.38/−0.71
Edf1	−0.44/−0.17	−0.40/−0.08	−0.51/−0.73	−0.78/−0.76	−0.81/−0.61	−0.26/−0.28	−0.26/−0.66
Id1	−0.98/−0.70	−0.97/−0.03	−0.74/−0.20	−0.99/−0.50	−0.95/−0.45	−0.27/−0.63	−0.66/−0.59
Manbal	−0.49/−0.51	−0.43/−0.43	−0.77/−0.34	−0.79/−0.19	−0.66/−0.19	−0.37/−0.20	−0.71/−0.25
Mocs3	−0.81/−0.53	−0.38/−0.02	−0.30/−0.90	−0.55/−0.95	−0.51/−0.95	−0.67/−0.55	−0.63/−0.57
Psmc3	−0.64/−0.46	−0.58/−0.20	−0.34/−0.29	−0.17/−0.58	−0.18/−0.36	−0.18/−0.67	−0.23/−0.93
Sdccag3	−0.44/−0.46	−0.48/−0.60	−0.76/−0.37	−0.77/−0.98	−0.74/−0.94	−0.73/−0.45	−0.67/−0.18
Slc39a13	−0.51/−0.59	−0.28/−0.77	−0.13/−0.42	−0.46/−0.73	−0.20/−0.75	−0.58/−0.72	−0.91/−0.80
Snai1	−0.84/−0.26	−0.37/−0.15	−0.92/−0.67	−0.93/−0.74	−0.95/−0.58	−0.95/−0.40	−0.97/−0.59
Spc25	−0.41/−0.37	−0.24/−0.40	−0.79/−0.91	−0.61/−0.91	−0.41/−0.91	−0.19/−0.58	−0.07/−0.85
Tubb2c	−0.73/−0.59	−0.84/−0.48	−0.47/−0.40	−0.74/−0.90	−0.77/−0.89	−0.73/−0.57	−0.81/−0.74
Srxn1	−0.49/−0.68	−0.71/−0.30	−0.32/−0.86	−0.96/−0.85	−0.93/−0.83	−0.69/−0.86	−0.41/−0.93
(B) Genes up-regulated between 1-and 4-folds in TCs as compared with other							
Dbndd2	−0.61/−1.00	−0.66/−0.98	−0.90/−0.73	−0.96/−0.96	−0.95/−0.96	−0.83/−0.87	−0.81/−0.90
Fbn1	−0.95/−0.98	−0.84/−0.53	−0.89/−0.84	−0.95/−0.82	−0.95/−0.84	−0.66/−0.87	−0.89/−0.76
Tfpi	−0.60/−0.72	−0.94/−0.58	−0.85/−0.85	−0.66/−0.95	−0.73/−0.96	−0.84/−0.78	−0.82/−0.86
Ak1	−0.80/−0.74	−0.84/−0.96	−0.69/−0.93	−1.00/−0.85	−0.97/−0.87	−0.94/−0.93	−0.97/−0.92
(C) Genes up-regulated between >4-folds in TCs as compared with others							
Myl9	−1.00/−0.96	−0.92/−0.91	−0.98/−0.92	−1.00/−0.96	−1.00/−0.98	−0.98/−0.88	−1.00/−0.94

**Table 2:** Summary of genes expressed preferentially in TCs, as compared with others

Compared pairs/fold down-regulated	>0	>1	>4
TC5 *versus* others	140	14	0
TC10 *versus* others	236	38	0
TCs *versus* others	80	6	0
Gene symbol	Folds (TC5 *versus* others/TC10 *versus* others)						
	Fibroblast	Stem	ATII	CD8_T_BL	CD8_T_LL	Basal_cell	Duct_cell
(A) Genes down-regulated between 0-and 1-folds in TCs as compared with others							
1500012F01Rik	1.00/2.24	2.51/4.68	0.09/0.29	0.83/1.11	1.30/1.68	2.50/3.01	4.17/4.95
1600027N09Rik	0.02/0.26	0.24/0.53	3.26/2.85	5.59/4.77	6.52/5.68	6.23/5.32	6.80/5.85
1700058C13Rik	0.01/0.29	0.03/0.31	6.00/5.54	6.63/5.93	15.2/13.91	7.23/6.46	4.82/4.30
2010317E24Rik	0.71/2.34	1.58/4.04	0.03/0.47	0.23/0.70	1.38/2.34	10.45/14.81	10.14/14.46
2810408M09Rik	1.13/1.13	0.66/0.66	20.58/14.77	1.23/0.58	14.91/10.44	65.85/46.34	29.46/20.69
4921504E06Rik	0.16/0.37	0.31/0.55	6.25/5.27	0.87/0.57	8.86/7.40	11.6/9.56	9.10/7.51
6820408C15Rik	0.02/0.06	0.54/0.61	99.87/75.87	8.76/6.22	28.01/20.77	26.11/19.03	17.2/12.51
Abca2	0.77/1.04	0.51/0.73	11.84/9.81	5.21/4.07	6.05/4.84	8.82/7.01	6.99/5.55
Acvr2a	1.31/1.33	0.42/0.43	23.28/16.86	13.98/9.7	25.29/18.05	84.04/59.65	70.93/50.56
Angptl2	0.52/0.45	1.97/0.31	16.78/0.65	9.96/1.22	5.33/2.03	14.94/3.34	20.00/1.38
Api5	0.06/0.28	0.40/1.50	3.39/9.94	6.09/5.56	4.92/2.84	3.10/8.51	3.11/11.60
Arhgap1	0.30/0.39	0.05/0.84	1.45/3.21	1.27/5.61	0.44/4.59	4.67/2.81	3.64/2.84
Arpc5l	1.84/1.17	0.11/0.75	1.81/1.99	9.73/1.70	7.56/0.73	9.55/5.73	5.43/4.53
Atf2	0.15/2.57	0.26/0.39	2.39/1.58	7.92/8.55	3.92/6.73	8.87/8.38	5.91/4.74
B2m	1.13/0.44	0.57/1.06	2.50/1.63	4.18/3.06	4.50/2.19	2.32/1.70	1.22/1.32
Catsper2	1.51/0.83	0.31/1.01	14.79/2.94	24.41/9.06	27.92/4.63	19.7/10.11	15.59/6.82
Cbfa2t2	0.21/0.82	0.43/0.34	0.27/1.18	0.97/2.13	0.86/2.37	0.60/1.00	0.28/0.35
Cdca7	1.89/1.49	6.28/0.30	1.05/10.46	1.2/16.91	0.10/19.66	2.18/13.55	2.49/10.73
Cep110	1.61/0.62	0.28/0.92	0.84/0.24	7.14/0.87	4.90/0.79	5.53/0.51	2.91/0.22
Chchd5	0.45/3.93	0.13/11.43	6.34/1.55	3.16/1.67	2.13/0.35	12.54/2.84	10.66/3.24
Ciz1	0.30/2.69	0.21/0.81	1.53/0.90	0.84/7.16	0.68/4.99	7.8/5.53	4.74/2.93
Cry2	0.11/1.18	0.15/0.69	13.7/7.05	4.83/3.43	2.42/2.38	10.9/13.38	11.47/11.46
Ddx27	0.42/0.74	0.44/0.62	0.20/1.47	1.27/0.74	1.09/0.61	5.47/7.32	6.52/4.46
Ddx31	0.31/0.36	0.93/0.91	0.51/2.65	1.79/8.07	0.75/9.21	5.42/17.28	5.09/13.41
Depdc7	1.34/0.33	0.42/0.38	13.2/11.89	0.67/3.96	2.85/1.96	2.96/9.11	33.03/9.65
Dsn1	0.61/0.26	1.33/0.31	6.39/6.62	39.29/12.48	47.16/10.54	12.99/6.54	23.88/5.26
Elp4	0.76/1.09	0.25/1.12	5.62/0.30	1.29/1.37	1.03/1.21	14.19/5.74	10.08/6.88
Ext2	0.44/0.46	0.68/1.16	23.82/0.23	31.23/1.21	21.21/0.41	20.92/4.07	11.20/3.83
Fmnl2	0.18/1.37	0.15/0.44	11.91/9.50	5.60/0.20	12.75/1.80	15.11/1.84	21.28/23.52
Fubp3	0.04/0.99	0.42/1.88	14.72/5.69	34.28/34.4	32.79/41.90	93.41/11.27	73.83/20.93
Gapvd1	0.04/0.18	0.09/3.47	3.56/5.04	5.04/13.37	3.84/8.91	3.82/4.01	3.24/2.43
Gmeb2	0.30/1.39	0.70/0.70	15.73/5.58	17.11/1.21	7.65/0.98	2.14/13.63	2.63/9.73
Hat1	0.56/0.99	0.09/1.32	12.93/24.06	49.03/30.61	46.29/21.08	26.19/20.45	29.7/11.00
Il15ra	1.42/0.59	0.07/0.54	2.25/11.67	0.64/5.29	2.21/12.29	4.80/14.33	1.58/20.32
Mapkbp1	0.03/0.18	0.13/2.25	96.09/4.23	25.15/33.87	22.48/30.68	123.55/25.01	263.85/15.93
Mga	0.68/0.24	0.34/0.68	4.24/12.63	4.57/28.72	5.14/27.86	8.89/78.35	7.84/62.23
Mkks	0.26/0.64	0.25/0.71	1.66/4.26	3.04/5.77	2.84/4.51	6.40/4.39	6.51/3.77
Mllt10	0.11/1.18	0.06/0.54	5.21/2.72	32.27/10.63	27.76/16.87	15.03/12.57	12.67/9.62
Mrps5	0.30/0.37	0.29/0.79	2.85/11.87	3.12/12.53	1.15/5.55	16.43/1.34	8.73/1.72
Necab3	3.74/0.72	0.49/0.20	3.72/10.21	2.23/38.12	6.28/36.48	11.85/20.21	3.01/23.07
Nr6a1	0.87/2.71	0.40/0.63	18.84/2.64	14.87/0.78	18.42/2.54	41.84/5.30	67.71/1.82
Ntng2	0.19/0.21	0.25/0.33	0.4/82.62	10.52/20.87	12.47/18.91	4.3/102.97	1.66/221.24
Nusap1	0.59/1.31	1.62/0.83	0.56/4.26	0.21/4.42	0.4/5.07	2.96/8.61	4.62/7.64
Olfr73	0.10/0.04	0.27/0.02	1.12/0.60	4.73/1.36	1.03/1.28	20.44/3.31	5.83/3.40
P2rx3	0.88/0.59	0.47/0.52	0.77/5.49	5.71/32.77	1.29/28.59	5.36/15.24	12.31/12.91
Pdhx	1.09/0.82	0.10/0.80	11.01/2.93	7.06/3.08	5.76/1.16	5.97/16.23	6.61/8.67
Phf20	0.77/3.35	0.87/0.37	0.88/2.17	5.00/1.11	3.20/3.81	1.03/7.36	0.67/1.62
Polr3f	0.83/1.25	0.21/0.69	10.77/16.44	10.86/12.56	14.35/15.82	15.73/35.52	7.5/57.87
Rae1	0.71/0.82	0.58/0.91	0.59/0.56	1.34/11.47	1.61/13.79	4.08/4.73	1.55/1.89
Rbm38	1.52/0.58	0.11/0.70	18.93/2.24	5.43/1.14	12.22/1.26	10.19/9.83	8.49/7.36
Scn1a	9.47/0.74	2.38/0.87	0.73/1.13	0.61/3.84	2.46/3.60	7.56/0.30	4.58/0.57
Slc12a5	4.24/0.92	0.59/2.15	2.84/0.38	3.26/0.04	3.41/0.21	4.93/2.38	5.08/3.83
Slc27a4	1.12/0.22	0.50/0.41	28.79/0.72	13.26/3.52	6.48/0.62	6.51/15.87	6.71/4.40
Slc34a3	0.33/1.15	0.38/0.69	10.17/0.48	10.98/4.46	19.71/0.89	20.08/4.17	17.79/9.87
Spata2	0.54/1.15	0.59/0.13	0.65/8.00	1.75/4.87	0.78/3.99	4.44/4.06	3.95/4.56
Sptlc3	0.42/0.74	0.31/1.10	26.19/5.08	2.31/7.18	13.32/8.05	179.36/10.23	250.13/8.40
Ss18l1	0.33/1.71	1.15/1.85	24.07/1.10	70.3/5.51	106.73/3.61	142.18/1.19	87.02/0.81
Surf6	0.14/2.78	0.03/0.71	0.36/0.10	1.56/1.44	0.98/0.59	4.86/3.33	5.65/2.12
Timm10	1.11/0.80	0.16/0.19	0.79/7.50	0.44/7.32	0.44/9.91	3.52/10.71	2.82/4.98
Trub2	0.41/1.52	0.58/1.33	0.34/0.72	1.71/1.45	0.55/1.77	4.38/4.31	1.61/1.68
Ttll9	0.06/2.02	0.03/0.34	13.59/16.46	4.75/4.47	8.44/10.41	63.39/8.51	39.11/7.1
Yme1l1	1.17/14.07	0.17/3.87	4.23/0.82	10.71/0.64	12.14/2.59	21.28/7.73	18.6/4.72
Arl6ip6	0.33/5.28	0.27/0.91	0.09/2.37	1.77/2.62	1.30/2.81	3.37/4.03	2.27/4.19
Cep152	0.58/2.53	1.26/5.71	1.95/0.32	28.84/1.17	18.63/0.56	5.03/3.14	2.63/3.27
Chd6	0.54/0.90	1.20/0.97	11.69/10.67	70.65/11.16	61.55/20.31	58.35/20.35	34.4/18.12
Ddb2	3.10/1.00	0.72/1.06	10.74/0.56	81.94/1.53	91.16/0.66	88.59/3.99	61.48/3.57
Dnmt3b	1.85/0.69	0.05/0.56	1.45/22.66	16.44/1.80	14.99/11.27	3.61/151.12	1.68/211.91
Dut	0.04/2.30	0.36/0.50	0.80/1.54	2.32/5.41	1.35/3.55	14.44/1.97	9.05/1.33
Emilin3	0.83/0.44	0.86/0.29	26.92/0.25	42.07/1.28	59.95/0.79	7.44/4.22	12.92/4.95
Entpd6	1.61/1.68	0.30/0.48	41.36/0.66	38.04/0.30	17.21/0.32	17.79/3.07	12.23/2.46
Mettl5	0.61/0.10	0.37/0.06	2.72/10.00	6.88/3.21	4.9/6.00	6.01/46.04	5.83/28.46
Myef2	0.58/0.69	0.11/0.33	0.74/1.38	0.91/3.04	2.12/3.57	4.87/9.78	3.94/8.22
Rif1	0.94/1.19	1.76/3.64	0.98/14.02	5.85/10.48	7.01/7.97	3.27/6.11	2.11/0.65
Sfmbt2	2.56/2.25	3.80/1.98	1.09/1.49	7.35/0.88	9.00/1.40	2.16/7.25	0.94/5.39
(B) Genes down-regulated between 1-and 4-folds in TCs as compared with others							
Gzf1	1/3.12	3.24/1.05	17.78/47.88	13.78/42.76	10.39/19.69	8.17/20.02	1.12/13.88
Pltp	9.94/3.83	2.17/3.01	189.9/16.60	7.67/31.99	8.30/21.33	110.19/13.79	96.66/22.44
Polr1b	1.83/1.59	1.33/1.47	7.01/5.03	6.45/10.25	3.06/18.41	10.71/37.36	8.50/46.25
Tasp1	2.19/7.78	1.12/8.13	1.96/2.59	15.91/2.34	12.47/4.49	20.97/8.59	8.31/9.50
Zbtb34	2.82/4.42	1.78/2.60	58.06/2.67	131.6/19.40	78.44/15.48	69.44/25.45	58.72/10.27
Zfp120	1.92/1.70	1.84/1.62	17.79/11.67	21.29/13.60	18.08/11.68	11.75/7.33	8.68/5.36

A set of genes are specifically up-or down-regulated in pulmonary TCs, as compared with other cells in chromosome 2 (Table3), up-or down-regulated genes more than 0-fold of TCs D5 were 576 or 341, 559 or 358, 228 or 689, 287 or 630, 277 or 640, 181 or 736, or 210 or 707, respectively, as compared with MSCs, Fbs, ATII, T-BL, T-L, ABCs, or PACs. Up-or down-regulated genes more than 0-fold of TCs D10 were 431 or 486, 408 or 509, 238 or 679, 294 or 623, 288 or 629, 182 or 735, or 222 or 695, as compared with MSCs, Fbs, ATII, T-BL, T-L, ABCs or PACs respectively. Up-and down-regulated genes more than 0-fold of TCs were 406 or 316, 388 or 338, 204 or 655, 262 or 598, 251 or 603, 158 or 712, or 180 or 665, as compared with MSCs, Fbs, ATII, T-BL, T-L, ABCs or PACs respectively.

**Table 3:** The number of genes specifically up-or down-regulated in pulmonary telocytes, as compared with other cells respectively

Compared pairs	Up>0	Up>1	Up>4	Down>0	Down>1	Down>4
TC5 *versus* stem	576	194	50	341	80	14
TC10 *versus* stem	431	136	41	486	152	27
TCs *versus* stem	406	116	30	316	73	14
TC5 *versus* fibroblast	559	201	79	358	107	17
TC10 *versus* fibroblast	408	166	61	509	178	33
TCs *versus* fibroblast	388	140	56	338	93	14
TC5 *versus* ATII	228	102	36	689	504	289
TC10 *versus* ATII	238	104	35	679	516	296
TCs *versus* ATII	204	86	30	655	476	268
TC5 *versus* CD8BL	287	174	89	630	689	303
TC10 *versus* CD8BL	294	196	97	623	485	284
TCs *versus* CD8BL	262	160	81	598	461	262
TC5 *versus* CD8LL	277	178	92	640	487	306
TC10 *versus* CD8LL	288	187	97	629	482	289
TCs *versus* CD8LL	251	162	84	603	458	263
TC5 *versus* basal cell	181	89	42	736	612	414
TC10 *versus* basal cell	182	101	41	735	601	406
TCs *versus* basal cell	158	79	36	712	573	375
TC5 *versus* duct cell	210	118	50	707	552	358
TC10 *versus* duct cell	222	117	51	695	548	345
TCs *versus* duct cell	180	103	42	665	522	320

In chromosome 3, 13 genes were higher than 0-fold in TCs, as compared with those in other cells (Table4), of which 10 genes (Agl, Ecm1, Golim4, Kcnab1, Lce1a2, Nexn, Pde4dip, Plekho1, Psrc1, Rhoc, Rit1, Scamp3, Sec22b) were overexpressed 0-to 1-fold (Table4A). Three genes Sh3glb1 (SH3-domain GRB2-like B1 – endophilin), Tm4sf1 (transmembrane 4 superfamily member 1) and Csf1 (colony stimulating factor 1) were overexpressed more than onefold, in both TC D5 and TC D10, as compared with other cells (Table4B). 59 genes in TCs were down-regulated, as compared with other cells (Table5). Of them, 1700013F07Rik, Amy1, Anp32e, Dnase2b, Fmo5, Pde5a, Phf17, Rwdd3 and Trim33 were down-regulated more than onefold, in both TC D5 and TC D10, as compared with other cells.

**Table 4:** Summary of genes expressed preferentially in TCs, as compared with others

Compared pairs/fold up-regulated	>0	>1	>4
TC5 *versus* others	42	10	0
TC10 *versus* others	30	7	2
TCs *versus* others	13	3	0
Gene symbol	Folds (TC5 *versus* others/TC10 *versus* others)						
	Fibroblast	Stem	ATII	CD8_T_BL	CD8_T_LL	Basal_cell	Duct_cell
(A) Genes up-regulated between 0-and 1-folds in TCs as compared with others							
Agl	−0.31/−0.25	−0.42/−0.37	−0.64/−0.6	−0.54/−0.5	−0.81/−0.79	−0.63/−0.6	−0.79/−0.77
Ecm1	−0.96/−0.96	−0.9/−0.9	−0.91/−0.91	−0.96/−0.96	−0.85/−0.85	−0.48/−0.47	−0.24/−0.23
Golim4	−0.65/−0.63	−0.24/−0.2	−0.86/−0.85	−0.8/−0.79	−0.86/−0.86	−0.65/−0.63	−0.48/−0.45
Kcnab1	−0.94/−0.95	−0.39/−0.54	−0.73/−0.8	−0.62/−0.71	−0.9/−0.93	−0.24/−0.42	−0.65/−0.73
Lce1a2	−0.16/−0.3	−0.41/−0.52	−0.01/−0.18	−0.03/−0.2	−0.24/−0.37	−0.83/−0.86	−0.93/−0.94
Nexn	−0.59/−0.47	−0.3/−0.08	−0.82/−0.76	−0.9/−0.87	−0.96/−0.95	−0.64/−0.52	−0.78/−0.71
Pde4dip	−0.65/−0.69	−0.52/−0.58	−0.19/−0.29	−0.11/−0.22	−0.4/−0.47	−0.71/−0.75	−0.68/−0.72
Plekho1	−0.07/−0.3	−0.8/−0.85	−0.8/−0.85	−0.85/−0.89	−0.97/−0.98	−0.95/−0.97	−0.98/−0.99
Psrc1	−0.8/−0.7	−0.78/−0.67	−0.81/−0.73	−0.72/−0.59	−0.62/−0.45	−0.32/0	−0.38/−0.1
Rhoc	−0.57/−0.52	−0.5/−0.43	−0.85/−0.83	−1/−1	−0.92/−0.91	−0.6/−0.55	−0.73/−0.69
Rit1	−0.54/−0.53	−0.66/−0.65	−0.58/−0.57	−0.69/−0.68	−0.66/−0.65	−−0.25/−0.22	−0.57/−0.55
Scamp3	−0.34/−0.34	−0.55/−0.55	−0.79/−0.79	−0.61/−0.61	−0.72/−0.72	−0.82/−0.82	−0.83/−0.83
Sec22b	−0.41/−0.08	−0.5/−0.23	−0.49/−0.21	−0.55/−0.3	−0.63/−0.43	−0.56/−0.32	−0.65/−0.46
(B) Genes up-regulated between 1-and 4-folds in TCs as compared with other							
Sh3glb1	−0.73/−0.68	−0.62/−0.54	−0.81/−0.77	−0.79/−0.75	−0.8/−0.76	−0.69/−0.62	−0.7/−0.63
Tm4sf1	−1/−1	−0.6/−0.67	−0.85/−0.88	−1/−1	−1/−1	−0.99/−0.99	−0.99/−0.99
Csf1	−0.71/−0.65	−0.73/−0.68	−0.93/−0.92	−0.98/−0.98	−0.98/−0.97	−0.91/−0.9	−0.92/−0.91

**Table 5:** Summary of genes expressed preferentially in TCs, as compared with others

Compared pairs/fold down-regulated	>0	>1	>4
TC5 *versus* others	79	12	0
TC10 *versus* others	137	22	1
TCs *versus* others	59	9	0
Gene symbol	Folds (TC5 *versus* others/TC10 *versus* others)						
	Fibroblast	Stem	ATII	CD8_T_BL	CD8_T_LL	Basal_cell	Duct_cell
(A) Genes down-regulated between 0-and 1-folds in TCs as compared with others							
1700027A23Rik	0.9/1.57	0.08/0.46	162.04/220.27	4.95/7.07	0.43/0.94	28.1/38.49	8.2/11.48
2810403A07Rik	0.81/1.11	0.7/0.99	0.47/0.72	3.33/4.07	4.16/5.04	1.31/1.7	0.96/1.3
4932438A13Rik	1.12/1.11	0.9/0.9	4.4/4.39	76.24/75.99	78.16/77.9	9.48/9.44	8.12/8.09
4933421E11Rik	1.61/2.74	0.22/0.74	0.3/0.87	5.03/7.64	4.6/7.02	2.41/3.89	0.14/0.64
A530020G20Rik	0.13/0.92	0.02/0.73	6.3/11.39	1.91/3.94	2.91/5.64	2.4/4.77	1.14/2.63
Acadm	5.66/5.92	0.22/0.27	4.5/4.72	6.8/7.1	4.87/5.09	6.25/6.53	5.1/5.33
Adh6a	0.46/0.67	0.44/0.65	5.68/6.63	8.32/9.65	11.57/13.37	50.97/58.39	25.27/29.03
Ahcyl1	0.62/0.88	0.14/0.32	1.5/1.91	1.14/1.49	0.51/0.76	2.4/2.95	1.9/2.38
Alx3	1.08/1.53	1.35/1.87	0.45/0.77	0.95/1.38	5.61/7.05	0.82/1.22	1.64/2.21
Atp11b	0.44/0.93	0.99/1.66	2.2/3.29	28.57/38.63	22.24/30.15	10.08/13.85	15.11/20.59
Car3	0.25/0.29	0.04/0.07	3.72/3.87	4.07/4.23	6.87/7.12	40.74/42.05	22.72/23.47
Clcc1	1.62/2.56	0.03/0.39	0.08/0.47	0.81/1.45	0.7/1.31	3.29/4.83	3.25/4.77
Cryz	3.65/6.13	3.67/6.15	0.91/1.92	0.17/0.79	0/0.53	0.51/1.32	2.14/3.8
Ctso	0.22/0.15	1.18/1.05	151.36/142.4	336.91/317.03	271.25/255.24	245.94/231.42	92.66/87.15
Gnat2	1.35/1.57	0.19/0.3	1.09/1.29	2.4/2.72	4.24/4.73	8.71/9.63	1.89/2.17
Gpsm2	1.89/2.66	1.39/2.03	0.36/0.72	3.42/4.59	5.15/6.78	14.01/17.99	18.95/24.24
Hax1	0.16/0.6	0.66/1.3	1.25/2.1	0.9/1.62	0.94/1.68	0.6/1.21	0.84/1.54
Hltf	0.31/0.45	0.45/0.61	3.26/3.75	18.55/20.78	18.24/20.44	7.82/8.83	3.57/4.09
Hps3	1.39/1.42	0.56/0.58	0.8/0.82	8.25/8.37	6.09/6.18	2.97/3.02	0.26/0.27
Ints3	0.83/0.94	0.23/0.3	27.92/29.65	13.8/14.68	6.12/6.54	23.06/24.5	26.27/27.9
Isg20l2	0.09/0.62	0.06/0.57	2.61/4.35	4.77/7.55	3.23/5.27	5.9/9.22	6.17/9.62
Lass2	0.06/0.12	0.01/0.07	5.54/5.9	2.13/2.3	1.64/1.79	1.12/1.23	0.41/0.49
Lrrc40	0.4/0.16	0.42/0.18	102.03/84.89	256.01/213.26	257.71/214.68	537.73/448.12	349.78/291.43
Lrrcc1	0.1/0.63	0.19/0.77	0.6/1.37	2.23/3.8	1.26/2.35	5.2/8.21	2.45/4.13
Mfsd8	0.71/0.3	0.45/0.11	11.71/8.67	24.91/18.72	19.66/14.72	32.59/24.56	33.42/25.19
Mrpl24	0.64/1.57	0.06/0.66	1.1/2.27	3.11/5.41	1.92/3.56	1.85/3.45	1.82/3.41
Mrpl9	0.29/0.47	0.44/0.64	3.16/3.73	7.18/8.3	5.33/6.19	8.23/9.48	7.87/9.08
Ndufb5	1/1.86	0.01/0.44	2.17/3.55	2.34/3.79	0.88/1.69	2.78/4.42	2.95/4.66
Odf2l	1.26/1.48	0.17/0.29	5.61/6.26	11.82/13.08	7.95/8.83	44.13/48.56	37.95/41.78
Papss1	1.13/1.25	0.44/0.52	2.44/2.62	1.53/1.66	0.28/0.34	1.73/1.88	1.64/1.78
Pgrmc2	1.11/1.55	0.08/0.31	4.75/5.96	1.45/1.97	2/2.62	29.73/36.17	15.37/18.8
Plk4	0.46/0.88	0.71/1.2	2.33/3.29	11.64/15.29	12.8/16.79	7.3/9.69	12.53/16.43
Prpf38b	1.18/0.93	0.68/0.49	11.25/9.81	50/44	66.2/58.3	44.75/39.37	34.78/30.58
Rabggtb	0.81/0.52	1.09/0.75	8.12/6.64	53.93/45.01	57.59/48.07	37.82/31.52	48.92/40.81
Rapgef2	0.29/0.68	0.63/1.11	2.8/3.92	10.47/13.85	8.15/10.86	0.24/0.6	0.61/1.09
Rps4x	0.11/0.67	0.62/1.45	0.39/1.1	0.85/1.79	1.99/3.51	0.67/1.53	0.54/1.32
Sars	3.16/4.85	1.32/2.27	1.03/1.86	0.4/0.98	0.09/0.54	0.41/0.99	0.3/0.83
Setdb1	0.25/0.87	0.6/1.39	0.03/0.55	1.29/2.42	1.07/2.1	2.73/4.58	1.8/3.19
Siah2	1.29/1.68	1.79/2.27	3.92/4.77	0.21/0.42	0.87/1.19	3.28/4.02	3.03/3.72
Slc33a1	0.75/0.58	0.72/0.55	5.56/4.91	2.19/1.87	1.75/1.48	3.02/2.62	0.75/0.58
Smc4	0.07/0.85	0.89/2.26	0.12/0.94	14.46/25.7	14.77/26.25	2.22/4.56	6.28/11.58
Sohlh2	0.02/0.11	0.08/0.17	1.69/1.93	0.91/1.07	1.86/2.11	1.58/1.8	1.85/2.1
Spata5	0.06/0.88	0.21/1.16	0.19/1.12	0.32/1.36	0.4/1.49	1.15/2.83	1.51/3.47
Syt6	0.23/0.6	0.21/0.57	0.15/0.5	0.55/1.02	0.43/0.87	1.27/1.96	0.29/0.69
Tbl1xr1	2.36/1.6	0.89/0.47	2.59/1.79	4.02/2.9	3.66/2.61	16.91/12.89	13.56/10.29
Txnip	0.77/0.21	1.35/0.6	54.04/36.58	107.86/73.33	65.79/44.6	36.39/24.53	26.15/17.53
Ubqln4	0.18/0.58	0.21/0.63	11.94/16.36	5.82/8.15	2.66/3.91	28.73/38.87	40.6/54.79
Wdr77	0.14/0.7	0.97/1.92	3.67/5.93	1.52/2.74	0.79/1.66	2.28/3.88	2.96/4.88
Ythdf3	0.22/0.54	0.64/1.07	3.08/4.17	6.8/8.87	6.44/8.41	3.68/4.92	3/4.06
Zzz3	0.1/0.64	0.73/1.58	0.1/0.64	3.91/6.33	3.67/5.96	1.29/2.42	0.6/1.39
(B) Genes down-regulated between 1-and 4-folds in TCs as compared with others							
1700013F07Rik	6.5/7.08	1.64/1.84	34.73/37.52	2.1/2.34	7.8/8.48	29.96/32.38	47.84/51.65
Amy1	2.81/3.86	8.06/10.55	9.44/12.32	7.37/9.67	14.79/19.13	31.07/39.88	24.64/31.68
Anp32e	8.69/11.5	2.79/3.89	121.76/157.32	245.2/316.52	243.57/314.42	356.1/459.55	464.63/599.52
Dnase2b	2.08/2.96	1.85/2.67	1.08/1.68	1.08/1.67	9.48/12.48	10.22/13.44	6.68/8.87
Fmo5	3.8/6	3.36/5.35	3.52/5.58	4.08/6.4	1.75/3.01	21.33/31.55	5.4/8.33
Pde5a	4.09/2.99	3.94/2.87	4.58/3.37	39.25/30.54	12.26/9.39	44.99/35.04	15.96/12.29
Phf17	1.24/1.76	3.48/4.52	1.04/1.52	3.4/4.42	3.67/4.75	11.3/14.14	4.48/5.75
Rwdd3	2.98/4.43	2.52/3.8	2.98/4.43	14.07/19.53	10.16/14.21	28.88/39.73	8.77/12.31
Trim33	1.93/5.56	1.06/3.62	4.2/10.64	26.9/61.48	17.78/41.07	3.1/8.17	2.63/7.13

In chromosome 3 (Table6), up-or down-regulated genes more than 0-fold of TCs D5 were 345 or 222, 352 or 215, 377 or 190, 214 or 353, 201 or 366, 130 or 437, or 137 or 430, as compared with Fbs, MSCs, ATII, T-BL, T-L, ABCs or PACs respectively. Up-or down-regulated genes more than 0-fold of TCs D5 were 265 or 302, 263 or 304, 138 or 429, 188 or 379, 168 or 399, 95 or 472, or 120 or 447, as compared with Fbs, MSCs, ATII, T-BL, T-L, ABCs or PACs respectively. Up-and down-regulated genes more than 0-fold of TCs were 255 or 212, 247 or 199, 367 or 128, 181 or 346, 164 or 362, 87 or 429, or 110 or 420, as compared with Fbs, MSCs, ATII, T-BL, T-L, ABCs or PACs respectively. Details of up-or down gene variations of chromosome 2 and 3, including the number and names of up-or down-regulated genes more than 0-fold among different cells, were listed in Data S2.

**Table 6:** The number of genes specifically up-or down-regulated in pulmonary telocytes, as compared with other cells respectively

Compared pairs	Up>0	Up>1	Up>4	Down>0	Down>1	Down>4
TC10 *versus* fibroblast	265	116	40	302	126	28
TC5 *versus* fibroblast	345	161	51	222	87	17
TCs *versus* fibroblast	255	100	33	212	78	12
TC10 *versus* stem	263	109	35	304	115	23
TC5 *versus* stem	352	134	41	215	63	12
TCs *versus* stem	247	85	27	199	58	12
TC10 *versus* ATII	138	67	22	429	306	177
TC5 *versus* ATII	377	278	137	190	93	32
TCs *versus* ATII	367	268	124	128	62	20
TC10 *versus* CD8BL	188	117	59	379	302	186
TC5 *versus* CD8BL	214	143	81	353	265	162
TCs *versus* CD8BL	181	113	57	346	254	153
TC10 *versus* CD8LL	168	99	51	399	307	197
TC5 *versus* CD8LL	201	138	66	366	278	163
TCs *versus* CD8LL	164	96	47	362	269	154
TC10 *versus* basal cell	95	55	19	472	388	261
TC5 *versus* basal cell	130	65	26	437	345	222
TCs *versus* basal cell	87	45	16	429	339	218
TC10 *versus* duct cell	120	56	26	447	373	234
TC5 *versus* duct cell	137	81	29	430	321	199
TCs *versus* duct cell	110	51	21	420	312	187

**Figure 1:**
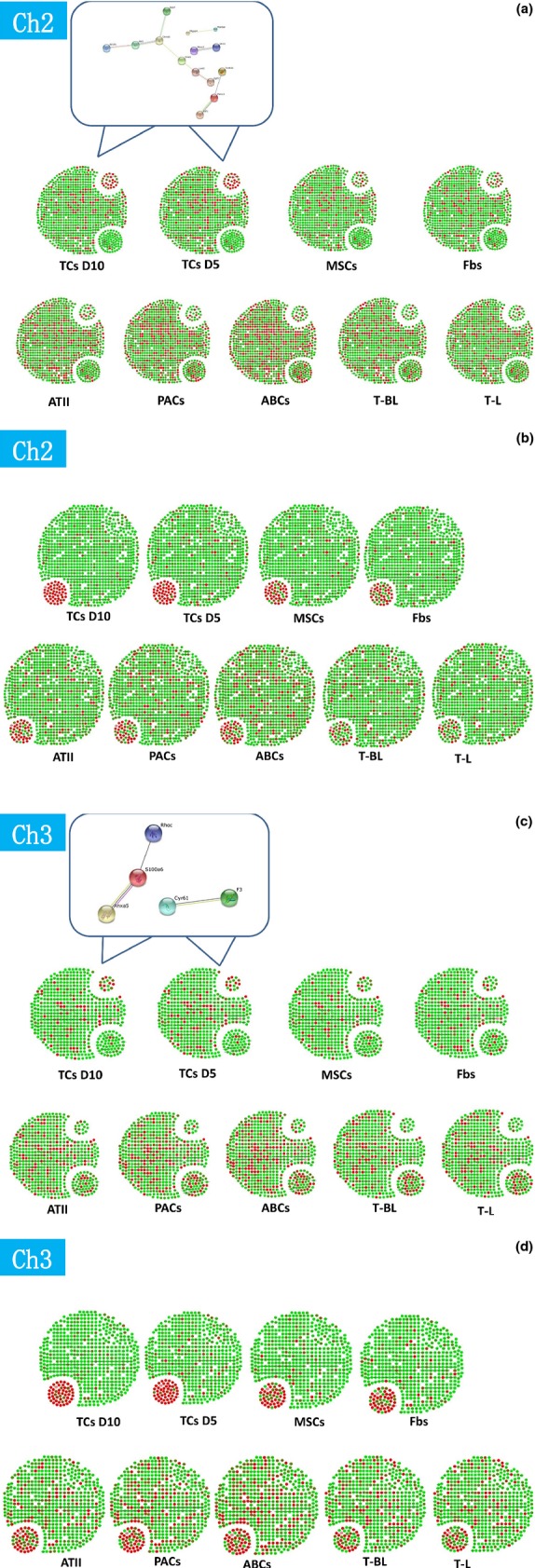
Expression profiles of the selected genes as an active group of chromosome 2 and 3 of TCs (TCs) isolated and cultured from mouse lungs on days 5 (D5) and 10 (D10), as compared with fibroblasts (Fbs), mesenchymal stem cells (MSCs), alveolar type II cells (ATII), airway basal cells (ABCs), proximal airway cells (PACs), CD8+ T cells come from bronchial lymph nodes (T-BL), and CD8+ T cells from lung (T-L) respectively (**A** and **C**). The profiles for entire genes are described in Data S1. The selected core network and whole mouse network are linked by the documented functional interactions from various databases (see Methods). Genes in each network are indicated in red and some of their nearest neighbours are indicated by dark grey nodes. A group of telocyte genes up-regulated and down-regulated more than 0-fold as compared with all other cells and existed in TCs on days 5 and 10 were selected as telocyte-specific or dominated genes in chromosome 2 and 3 (**A** and **C**). Top 50 up-or down-regulated genes of each cells were also evaluated and their distribution within chromosome 2 and 3 genes showed the difference between cells (**B** and **D**). Details of the selected network in each cell type are in Figure S1–S18.

The relationships of the more than 0-fold up-regulated genes of chromosome 2 and 3 in TC D5 and/or TC D10 were analysed by String Network analysis (www.string-db.org), as compared with other cells, to identify direct (physical) and indirect (functional) associations between selected genes of TCs. TC-specific or dominating genes in TC D5 and TC D10 were selected by up-or down-expression more than 0-fold, as compared with other cells. Figure1A and C demonstrated the distribution of such active gene group in chromosome 2 and 3 of all cells, and interactions or potential functional links of those genes of TCs.

In chromosome 2, about 30-50% of TCs genes showed similar patterns of gene expression in MSCs, Fbs or ATII, while 5-15% of TCs genes showed similarities with ABCs, PACs, T-BL or T-L. Top 50 up-or down-regulated genes of each cell were also evaluated and their distribution within chromosome 2 genes showed the difference between them, as shown in Figure1B. High expressed genes of each cell within chromosome 2 were evaluated and distributed as red colour (Fig.1B). The distribution of the high expressed genes and low expressed genes both in TC D5 and TC D10 indicates that they are in the centre of the small cluster and different from the other cells. Among the 26 co-up-expressed genes (Table1A–C), 7 genes were found to have certain interactions (Fig.1A).

**Figure 2:**
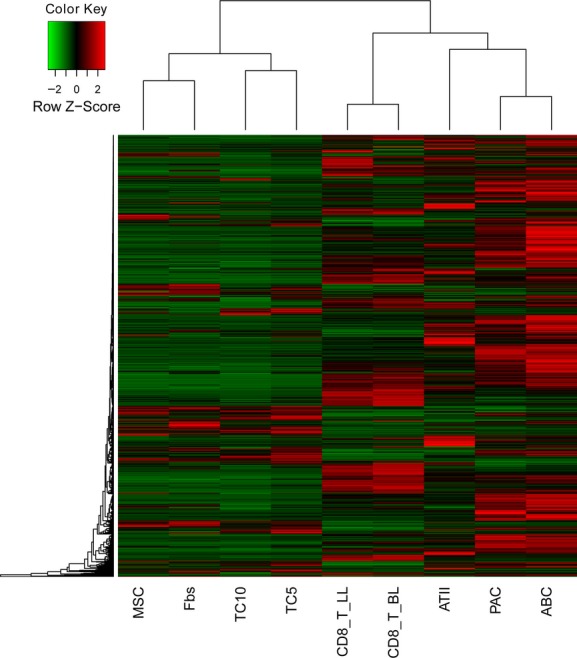
Hierarchical cluster analysis of the differentially expressed genes on chromosomes 2 and 3 among telocytes (TCs), mesenchymal stem cells (MSCs), fibroblasts (Fbs), lymphocytes from lungs (T-LL) and from bronchial lymph nodes (T-BL), alveolar type II cells (ATII), proximal airway cells (PAC) and airway basal cells (ABC).

In chromosome 3, about 50–60% of TCs genes showed similar patterns of gene expression in Fbs, MSCs, PACs or ABCs, while 0–20% of TCs genes showed similarities with ABCs, PACs, T-BL or T-L. Top 50 up-or down-regulated genes of each cell were also evaluated and their distribution within chromosome 3 genes showed the difference between them, as shown in Figure1D. High expressed genes of each cell within chromosome 3 were evaluated and distributed as red colour (Fig.1D). The distribution of up-expressed genes and down-expressed genes in TC D5 and TC D10 indicates that they are in the centre of the small cluster and different from the other cells. Among the 16 co-up-expressed genes (Table4A and B), no clear or certain interactions (Fig.1C) were found. The hierarchical cluster analysis of the differentially expressed genes (Fig. 2) clearly shows that TCs are poorly related to the other cell lines.

## Discussion

Mouse chromosomes are the best studied mammalian chromosomes and are considered as gold standard of human comparative map, although genomic rearrangements occur during evolution. Certain human disease genes were discovered by comparative genomics using the information derived from mapped mouse mutations, although they are not the simplest model for human comparison. In humans, chromosome 2 has the largest sequenced base pairs (237, 712, 649) 47, working with all of the autosomes in humans, spanning the second largest amount of total base pairs (242, 751, 149) and representing 16.2% of the total DNA 48–51. Over 31 exactly known diseases were proposed to be associated with genes on chromosome 2. In mouse, chromosome 2 is entirely sequenced and has 3146 genes encoding 1780 proteins 47, of which 917 genes were measured by bioinformatics tools in the present study. Our data demonstrated that there were 26 or 80 up-or down-regulated genes of chromosome 2 in TCs, as compared with MSCs, Fbs, ATII, T-BL, T-LL, ABCs or PACs.

One gene Myl9 (myosin, light polypeptide 9) was overexpressed most in TCs, different from other cells. Myl9 regulatory gene encodes the regulatory light chains of myosin II molecule, known to play a central role in cell adhesion, migration and division. Recent results showed Myl9 as the only gene differentially expressed in the aged *versus* young injured arteries 52 implying that it may be related to tissue/organ injury and ageing. Therefore, it is possible that the overexpression of Myl9 in pulmonary TCs may play an important role in lung injury and ageing. There were six genes, *e.g*. Pltp, Gzf1, Polr1b, Tasp1, Zbtb34 and Zfp120, down-expressed most in TCs, different from other cells. The Pltp (phospholipid transfer protein) gene is widely expressed in the body, and plays an important role in lipid metabolism, immune modulation, lipopolysaccharide binding or neurodegenerative disease 53. Pltp is highly expressed within the lung epithelium, in chronic obstructive pulmonary disease or pulmonary inflammation 54. TCs may play an important role of inhibiting inflammation in the lung. Roles of Gzf1 (GDNF-inducible zinc finger protein 1), Polr1b (polymerase (RNA) I polypeptide B), Tasp1 (taspase threonine aspartase 1), Zbtb34 (zinc finger and BTB domain containing 34) or Zfp120 (zinc finger protein 120) genes or proteins in the lung remain unclear. Thus, there is a further need to clarify the exact mechanisms and functions of these genes in TCs.

Mouse chromosome 3 has a total number of genes of 993 which encode a total of 669 proteins 47. Human chromosome 3 has ∼7% of the human genome probably related with, at least, 121 diseases that are associated with 105 genes 55 and also spanning the third largest amount of total base pairs (199, 446, 827) and represented about 6.5% of the total DNA in cells 56–59. The chromosome 3 has 1550 genes, of which 567 genes of chromosome 3 were measured by bioinformatics tools in the present study. We showed that there were 13 or 59 up-or down-regulated genes of chromosome 3 in TCs, as compared with Fbs, MSCs, ATII, T-BL, T-L, ABCs or PACs. There were three genes, *e.g*. Sh3glb1, Tm4sf1 and Csf1, overexpressed in TCs.

Sh3glb1 gene encodes SH3-domain GRB2-like B1 or endophilin, known to have an extremely close relationship with Bax-interacting factor-1 (bif-1) 60,61, involved in cell survival and proliferation under metabolic stress and evasion of apoptosis. SH3glb1 is a membrane curvature-inducing protein interact with BECN1 though UVRAG and regulates the post-Golgi trafficking of membrane-integrated ATG9A for autophagy. At the premalignant stage, allelic loss of Sh3glb1 could enhance Myc-induced chromosomal instability and result in the up-regulation of anti-apoptotic proteins, including MCL1 and BCL2L1 61, being responsible for enabling cells to survive and proliferate under metabolic stress and evasion of apoptosis. Endophilin is a membrane curvature-inducing protein that interacts with autophagy related beclin 1, although UV radiation resistance associated gene (Uvrag) and regulates the post-Golgi trafficking of membrane-integrated autophagy related 9A (Atg9A) protein. At the premalignant stage, allelic loss of Sh3glb1 enhances Myc-induced chromosomal instability and results in the up-regulation of anti-apoptotic proteins, including MCL1 and BCL2L1 61. So far, there is no reported association with any lung disease, however, we cannot exclude a role for TCs as having pro-proliferative effects through inhibition of apoptosis as showed in a previous study 41.

Tm4sf1 (transmembrane 4 superfamily member 1) is a distant member of the transmembrane 4 superfamily of cell-surface proteins characterized by the presence of four hydrophobic domains 62. It is highly expressed in different carcinomas, *e.g*. in lung cancer 62, and lowly expressed in normal tissues 63. Colony stimulating factor 1 (macrophage) (Csf1) plays an important role in cancer metastasis and invasion. It is highly expressed in different carcinomas and expressed at relatively low levels (if at all) in many normal tissues 63. High expression of Csf1 can increase metastasis and invasion of pulmonary adenocarcinomas 64. For example, Tm4sf1 it was up-regulated in human adenocarcinoma A549 cell line, suggesting a poor prognosis for anticancer therapy 65. Overexpression of TM4SF1 and Csf1 in lung TCs may have a role in the development of lung cancer. Among down-expressed genes in TCs, Pde5a (cGMP-specific phosphodiesterase 5A) has an obvious association with acute and chronic interstitial lung disease. Overexpression of Pde5a may accelerate the formation of pulmonary fibrosis, while down-expression of Pde5a has important roles and effects in pulmonary fibrosis-associated pulmonary hypertension 66,67. Therefore, we concluded that Tm4sf1 and Csf1 found to be overexpressed in lung TCs may have a role in tumour promotion. There were nine genes, *e.g*. 1700013F07Rik, Amy1, Anp32e, Dnase2b, Fmo5, Pde5a, Phf17, Rwdd3 and Trim33, down-expressed most in TCs, different from other cells.

Among them, only phosphodiesterase 5a (Pde5a) cGMP-specific gene is obviously associated with acute and chronic interstitial lung disease. Its high expression promotes the pulmonary fibrosis, while the inhibition of Pde5a expression ameliorates right ventricular failure and pulmonary, when is associated with bleomicin, through a reduction in reactive oxygen species 68. Therefore, Pde5a low expression in lung TCs may have therapeutic effect on pulmonary fibrosis and other acute and chronic interstitial lung disease, probably by modulation of oxidative stress levels, as previously shown 41.

In conclusion, the present study compared genetic variations of chromosome 2 and 3 of pulmonary TCs with other related cells, *e.g*. Fbs, MSCs, ATII, T-BL, T-L, ABCs or PACs. Our data showed a number of TCs-specific or dominant genes in chromosomes 2 and 3, different from other lung tissue resident cells or infiltrated cells. The TCs signatures of chromosome 2 and 3 genes indicate TCs may be mainly associated with anti-inflammatory responses, the prevention of lung cancer formation and development or protective effects on pulmonary fibrosis or acute and chronic interstitial lung diseases.

## Supporting Information

Data S1 Data profiles for all genes.

Data S2 Details of up-or down regulated gene expression variations of chromosome 2 and 3.

Figure S1 Details of the selected core network genes in TCs isolated from the mouse lung and cultured for 5 days in chromosome 2.

Figure S2 Details of the selected core network genes in TCs isolated from the mouse lung and cultured for 10 days in chromosome 2.

Figure S3 Details of the selected core network genes in mouse mesenchymal stem cells in chromosome 2.

Figure S4 Details of the selected core network genes in mouse fibroblasts in chromosome 2.

Figure S5 Details of the selected core network genes in mouse alveolar type II cells in chromosome 2.

Figure S6 Details of the selected core network genes in mouse airway basal cells in chromosome 2.

Figure S7 Details of the selected core network genes in mouse proximal airway cells in chromosome 2.

Figure S8 Details of the selected core network genes in mouse CD8^+^ T cells come from bronchial lymph nodes in chromosome 2.

Figure S9 Details of the selected core network genes in mouse CD8^+^ T cells from lung in chromosome 2.

Figure S10 Details of the selected core network genes in TCs isolated from the mouse lung and cultured for 10 days in chromosome 3.

Figure S11 Details of the selected core network genes in TCs isolated from the mouse lung and cultured for 5 days in chromosome 3.

Figure S12 Details of the selected core network genes in mouse mesenchymal stem cells in chromosome 3.

Figure S13 Details of the selected core network genes in mouse fibroblasts in chromosome 3.

Figure S14 Details of the selected core network genes in mouse alveolar type II cells in chromosome 3.

Figure S15 Details of the selected core network genes in mouse airway basal cells in chromosome 3.

Figure S16 Details of the selected core network genes in mouse proximal airway cells in chromosome 3.

Figure S17 Details of the selected core network genes in mouse CD8^+^ T cells come from bronchial lymph nodes in chromosome 3.

Figure S18 Details of the selected core network genes in mouse CD8^+^ T cells from lung in chromosome 3.
