# Supplementary figures and images for: Variations of chromosomes 2 and 3 gene expression profiles among pulmonary telocytes, pneumocytes, airway cells, mesenchymal stem cells and lymphocytes
Source: J Cell Mol Med. 2014 Oct 2;18(10):2044–60. doi: 10.1111/jcmm.12429 (PMC4244019; doi:10.1111/jcmm.12429)

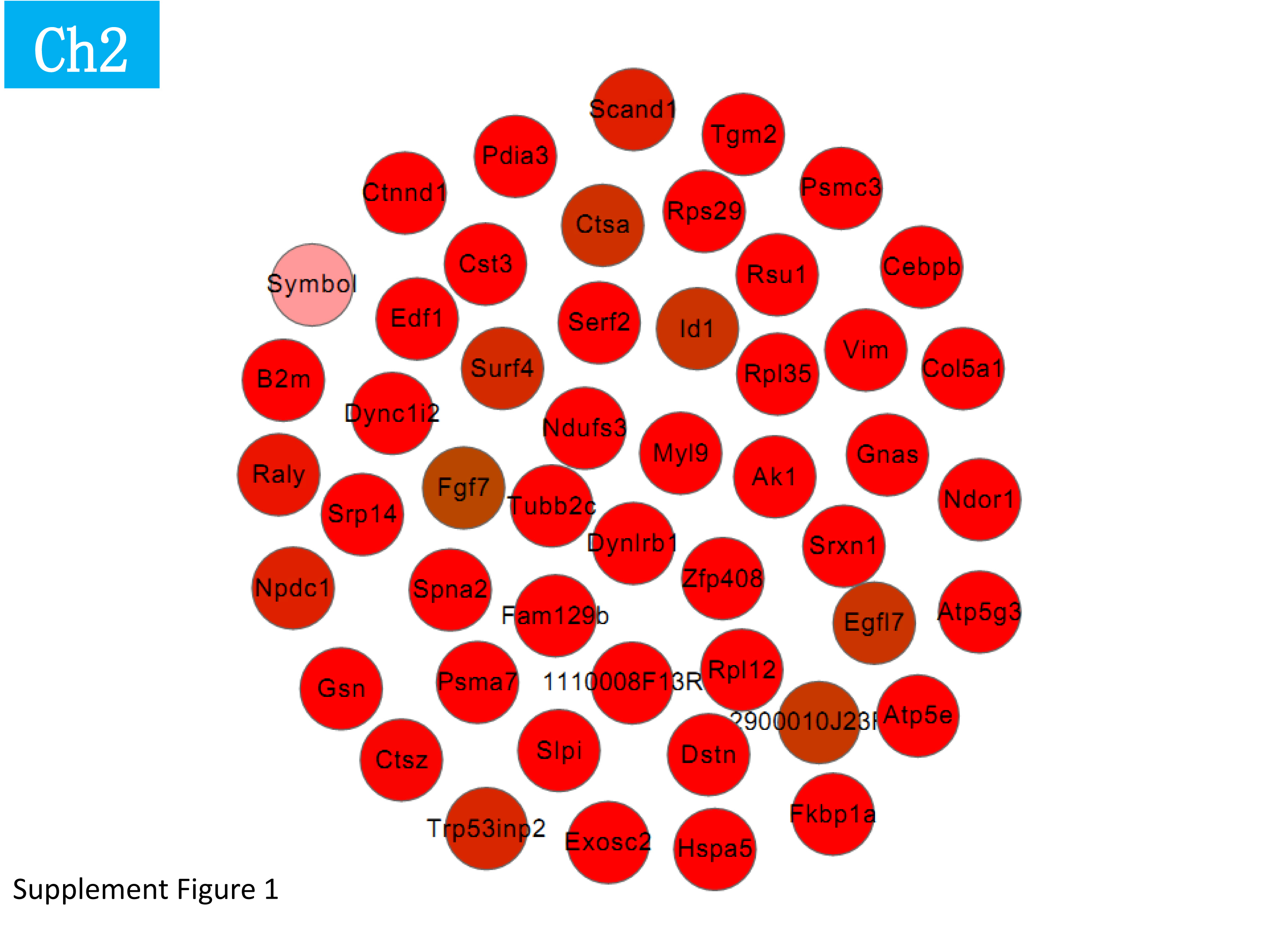

Supplement: Figure S1 — Details of the selected core network genes in TCs isolated from the mouse lung and cultured for 5 days in chromosome 2. [file jcmm0018-2044-sd3.jpg]

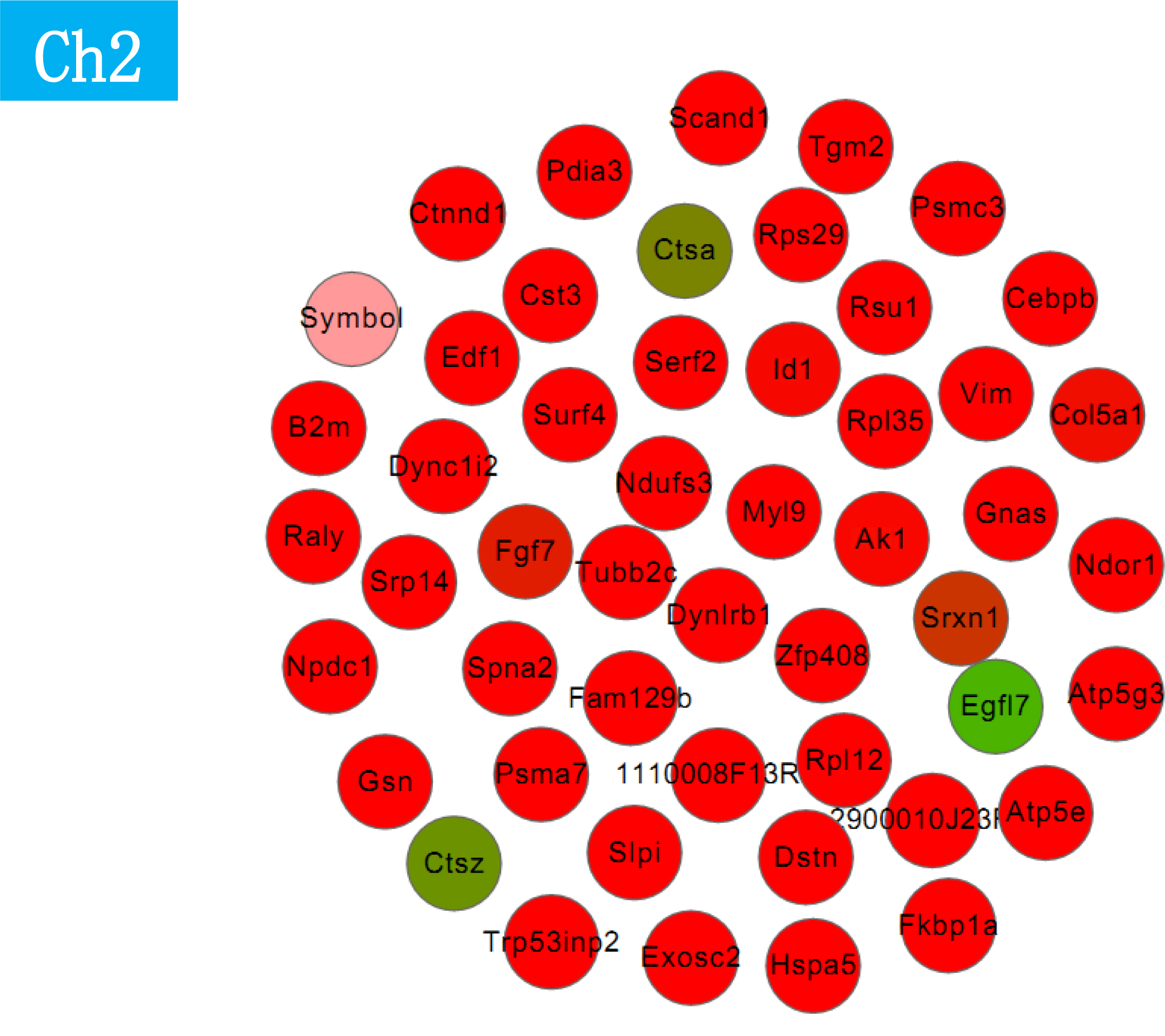

Supplement: Figure S2 — Details of the selected core network genes in TCs isolated from the mouse lung and cultured for 10 days in chromosome 2. [file jcmm0018-2044-sd4.jpg]

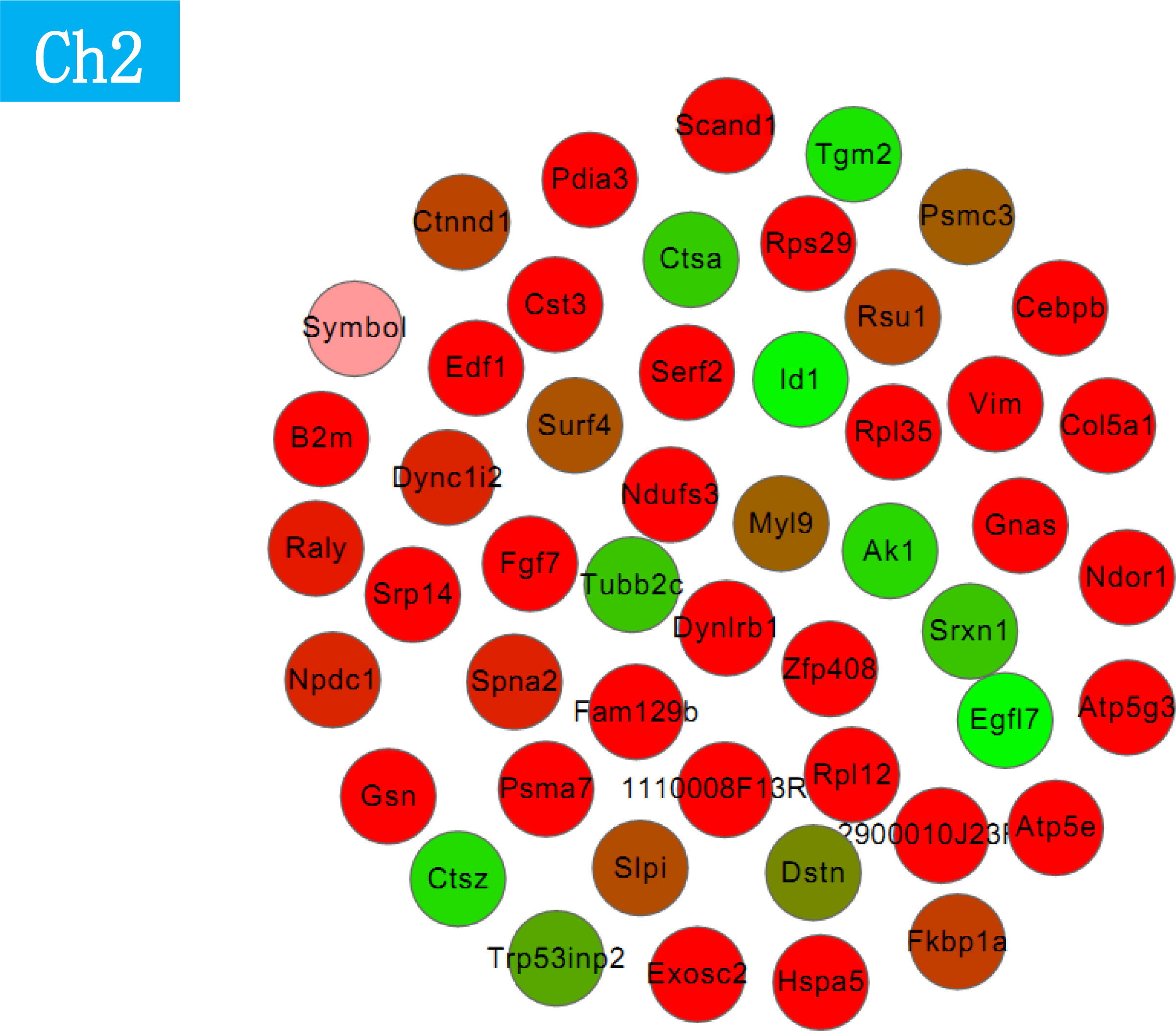

Supplement: Figure S3 — Details of the selected core network genes in mouse mesenchymal stem cells in chromosome 2. [file jcmm0018-2044-sd5.jpg]

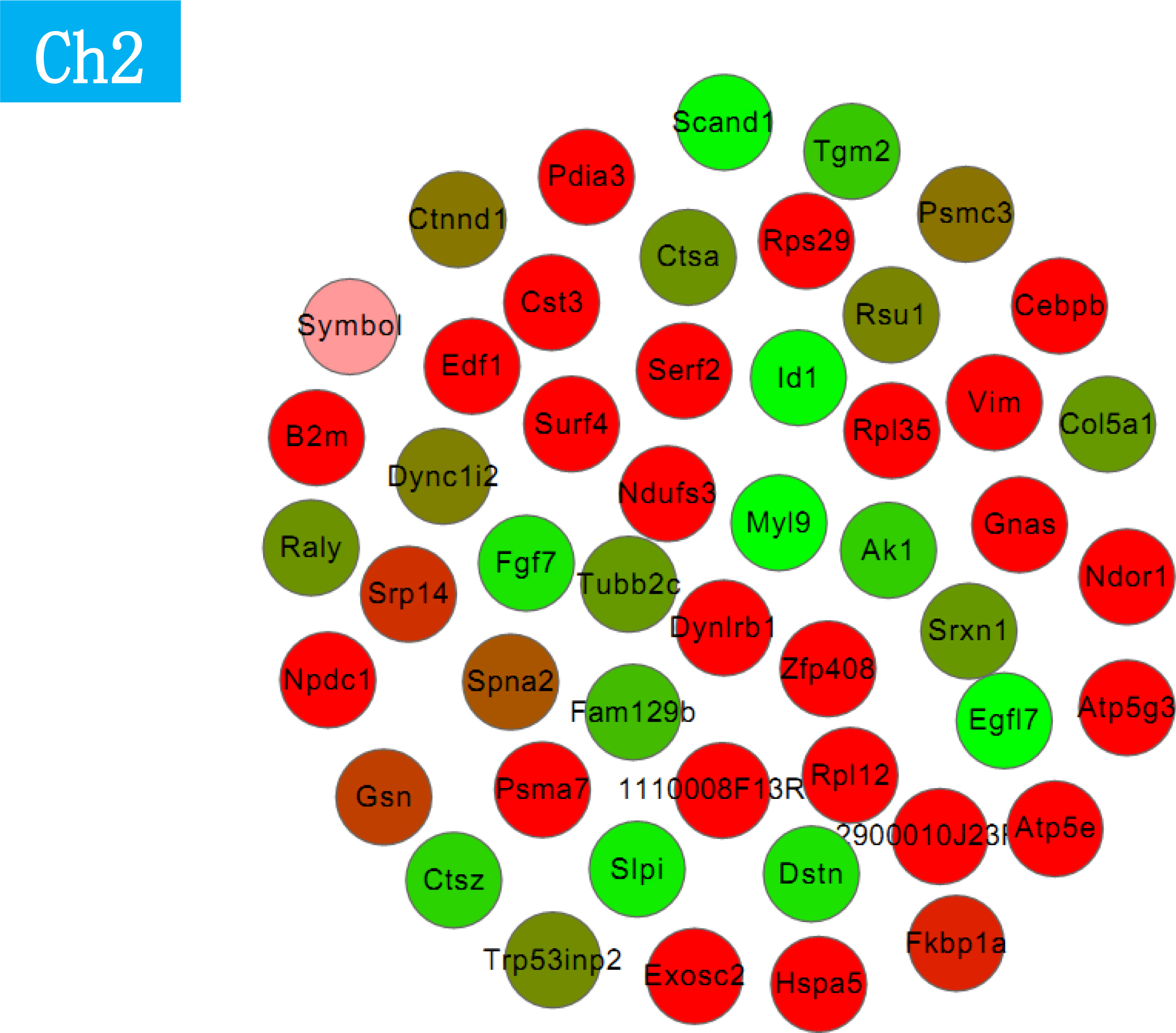

Supplement: Figure S4 — Details of the selected core network genes in mouse fibroblasts in chromosome 2. [file jcmm0018-2044-sd6.jpg]

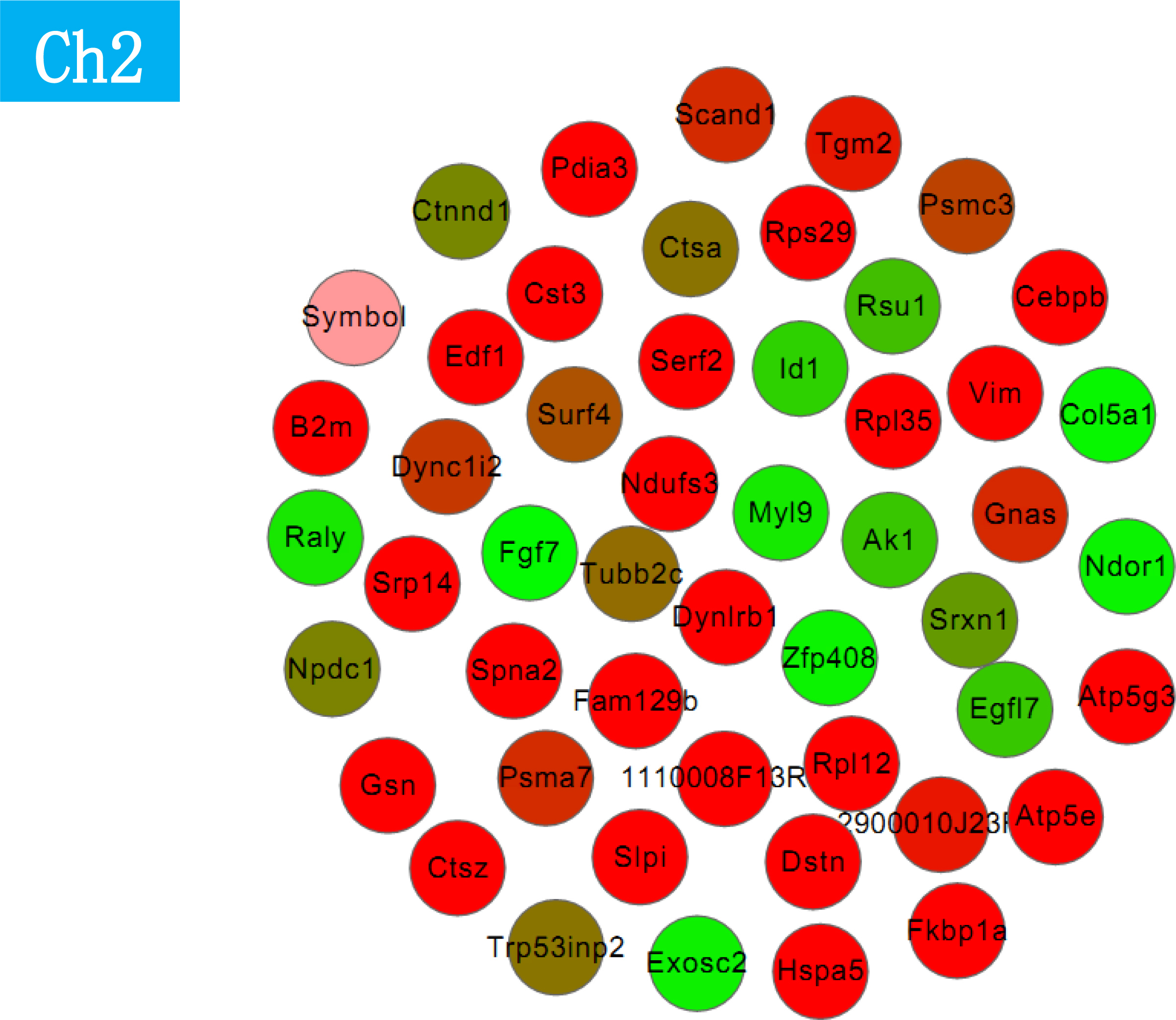

Supplement: Figure S5 — Details of the selected core network genes in mouse alveolar type II cells in chromosome 2. [file jcmm0018-2044-sd7.jpg]

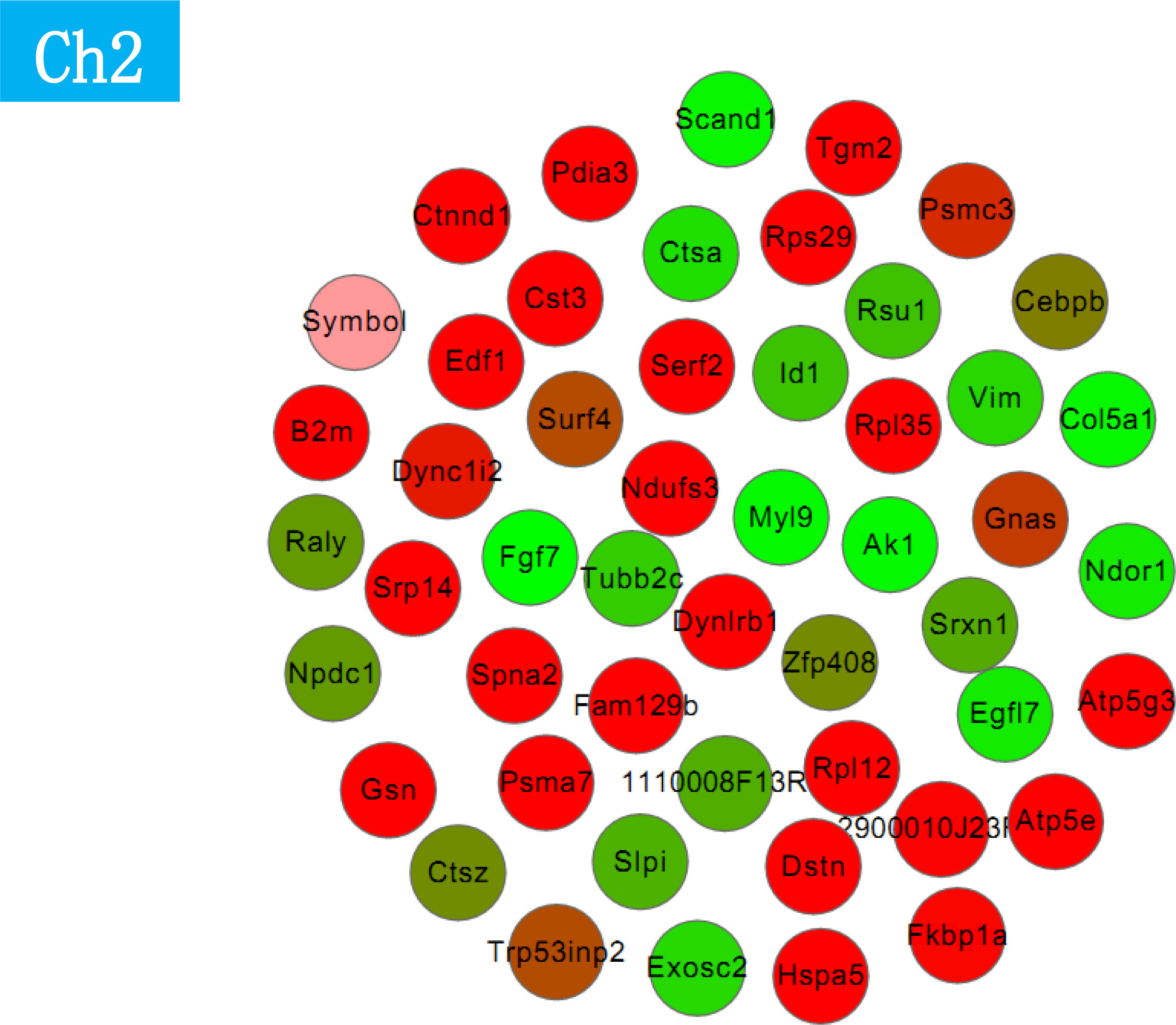

Supplement: Figure S6 — Details of the selected core network genes in mouse airway basal cells in chromosome 2. [file jcmm0018-2044-sd8.jpg]

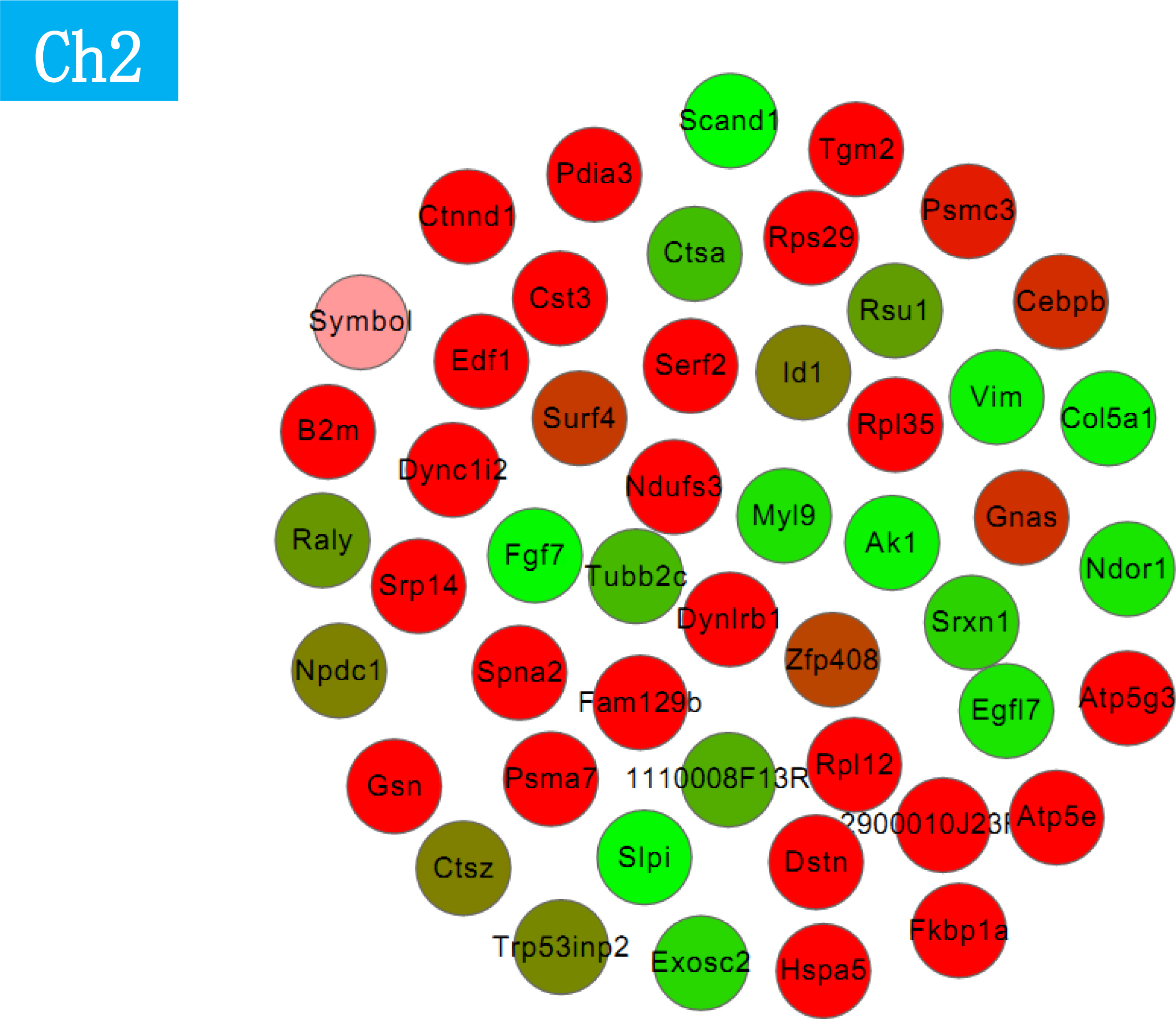

Supplement: Figure S7 — Details of the selected core network genes in mouse proximal airway cells in chromosome 2. [file jcmm0018-2044-sd9.jpg]

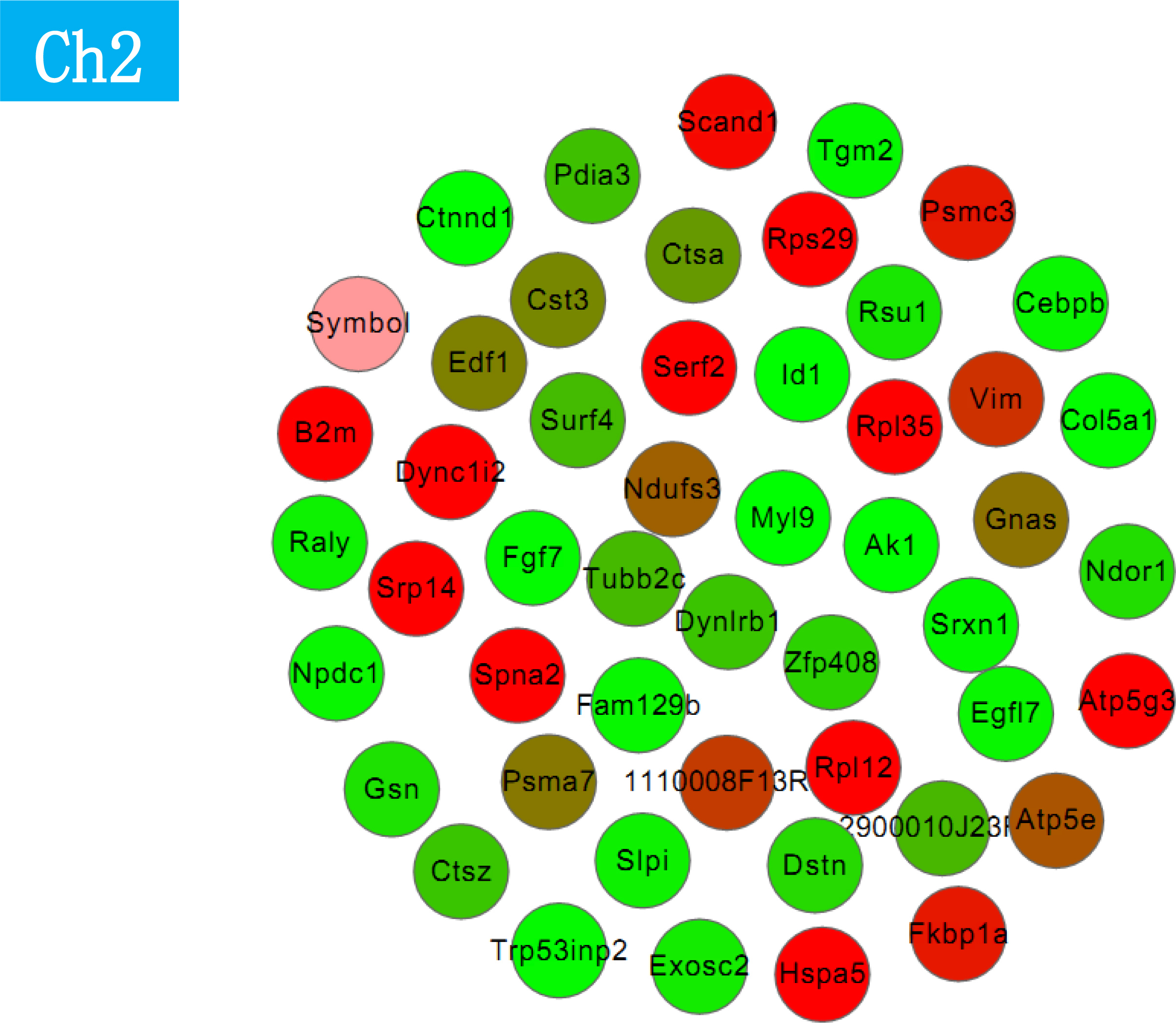

Supplement: Figure S8 — Details of the selected core network genes in mouse CD8+ T cells come from bronchial lymph nodes in chromosome 2. [file jcmm0018-2044-sd10.jpg]

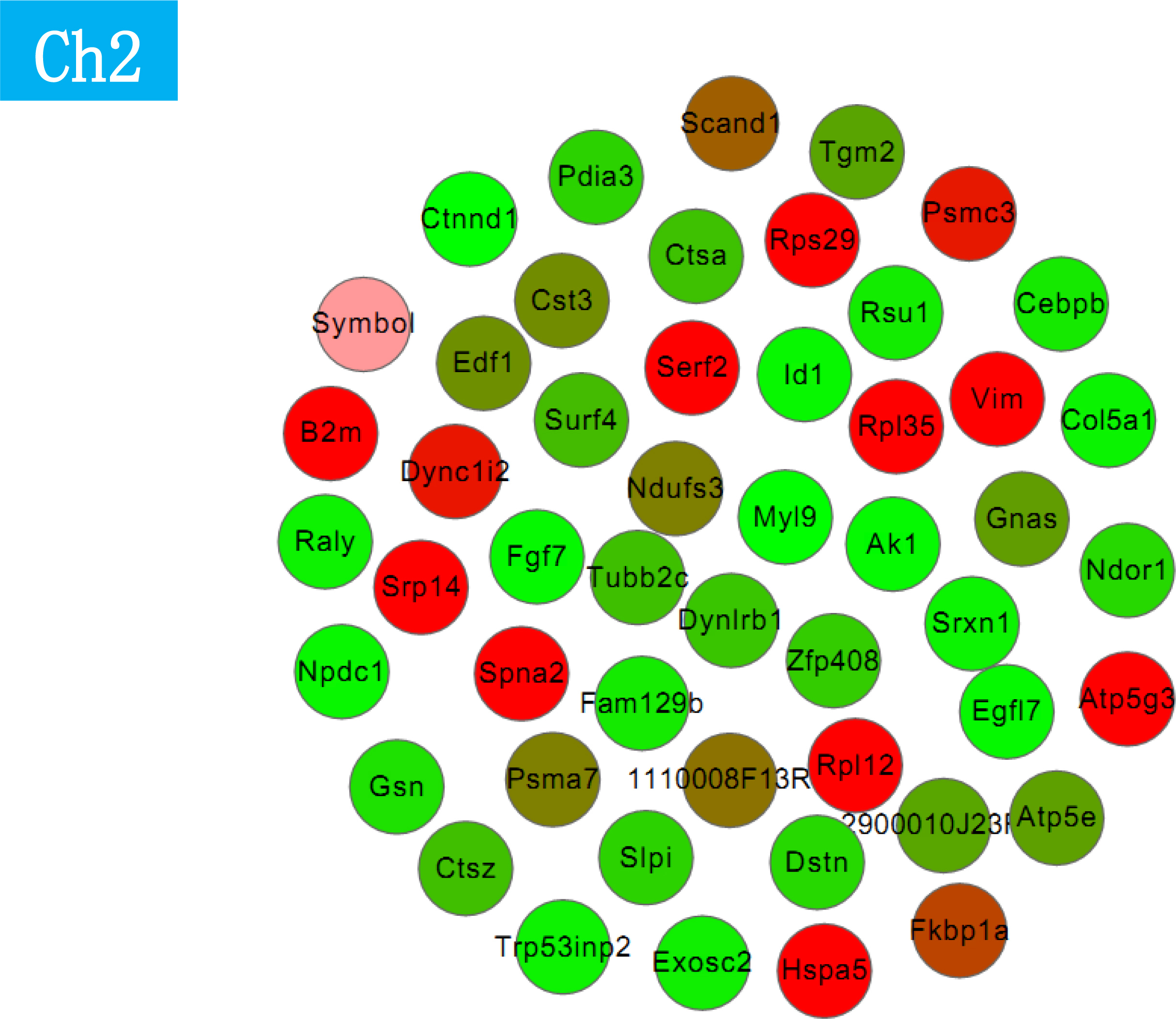

Supplement: Figure S9 — Details of the selected core network genes in mouse CD8+ T cells from lung in chromosome 2. [file jcmm0018-2044-sd11.jpg]

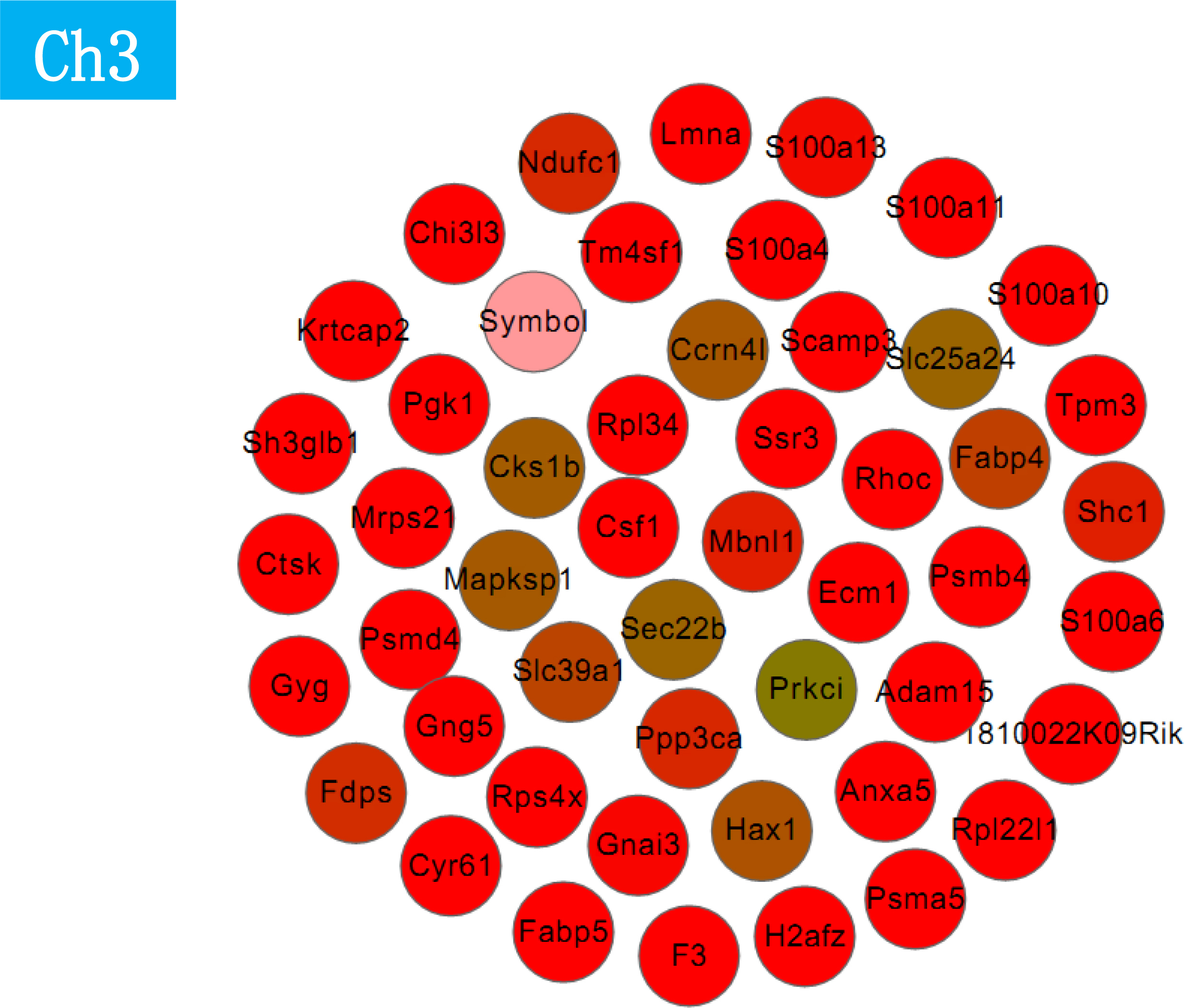

Supplement: Figure S10 — Details of the selected core network genes in TCs isolated from the mouse lung and cultured for 10 days in chromosome 3. [file jcmm0018-2044-sd12.jpg]

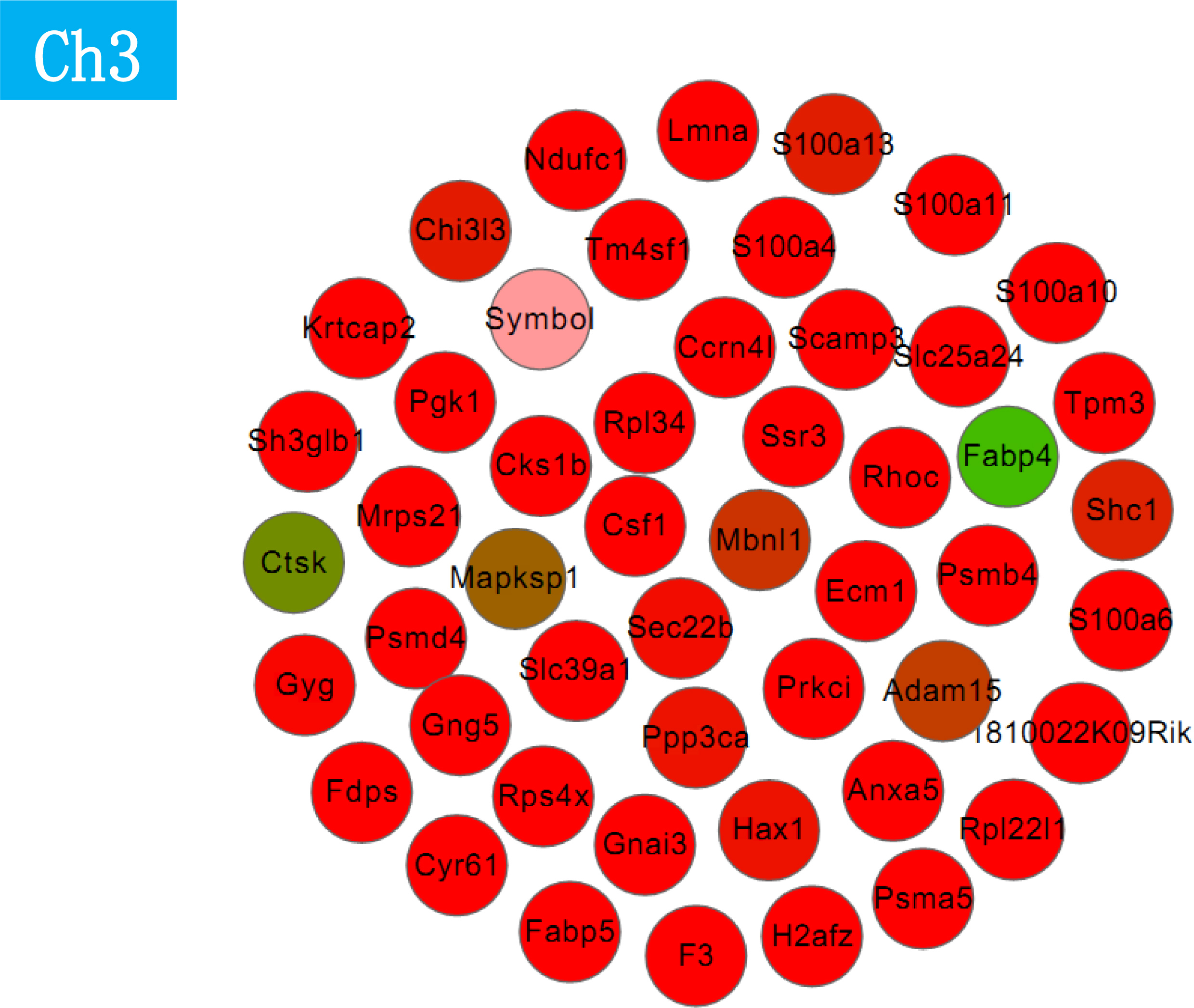

Supplement: Figure S11 — Details of the selected core network genes in TCs isolated from the mouse lung and cultured for 5 days in chromosome 3. [file jcmm0018-2044-sd13.jpg]

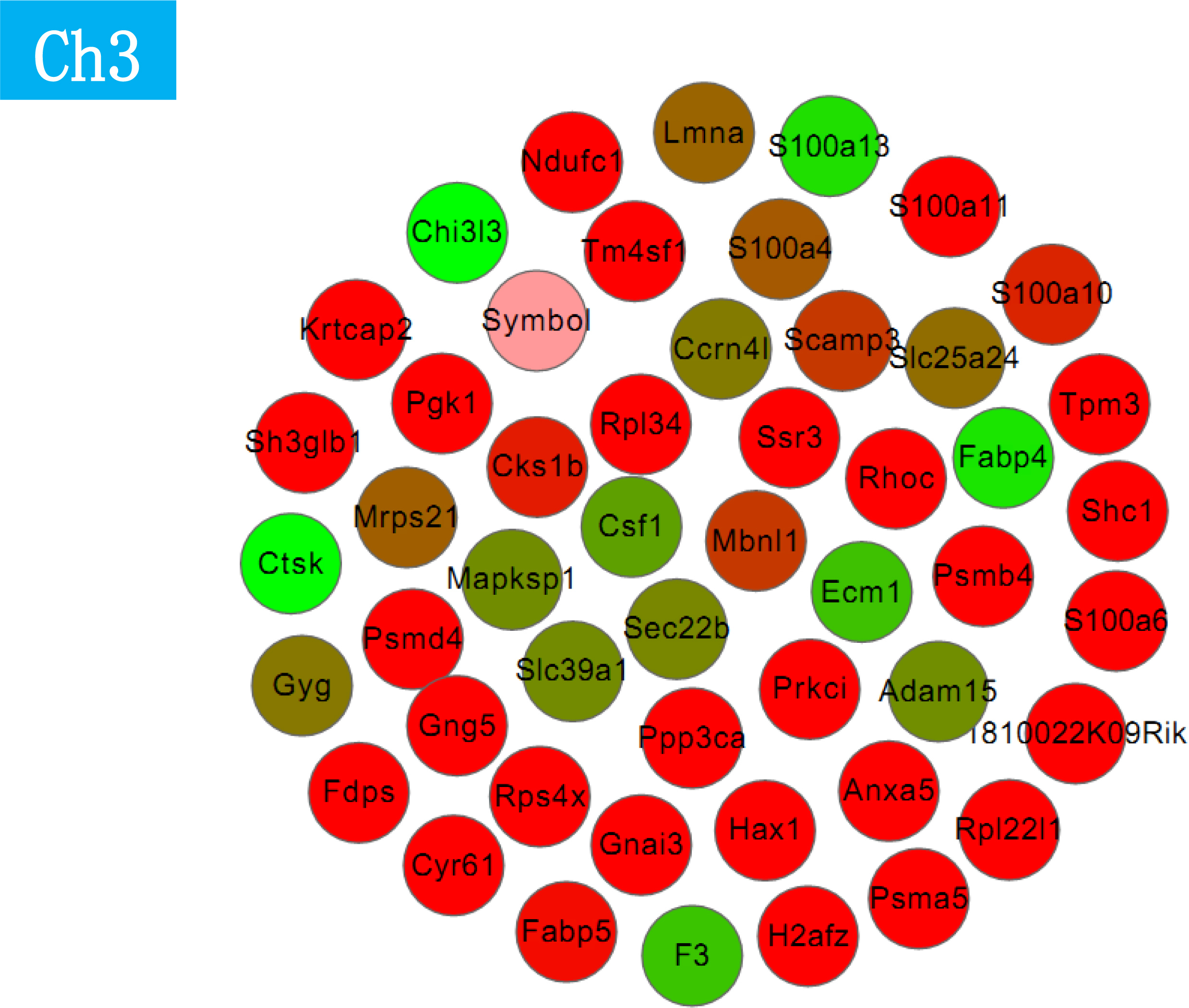

Supplement: Figure S12 — Details of the selected core network genes in mouse mesenchymal stem cells in chromosome 3. [file jcmm0018-2044-sd14.jpg]

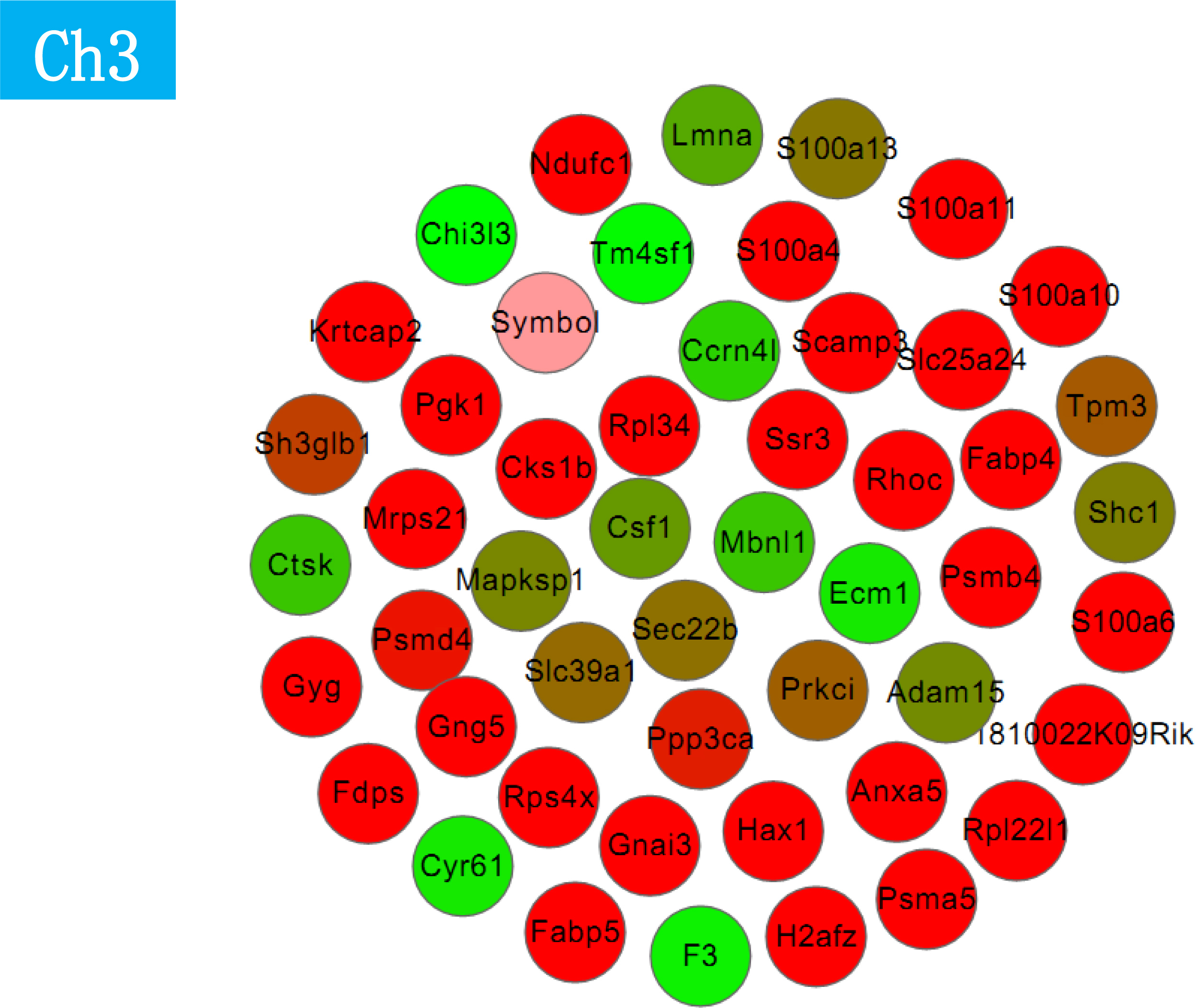

Supplement: Figure S13 — Details of the selected core network genes in mouse fibroblasts in chromosome 3. [file jcmm0018-2044-sd15.jpg]

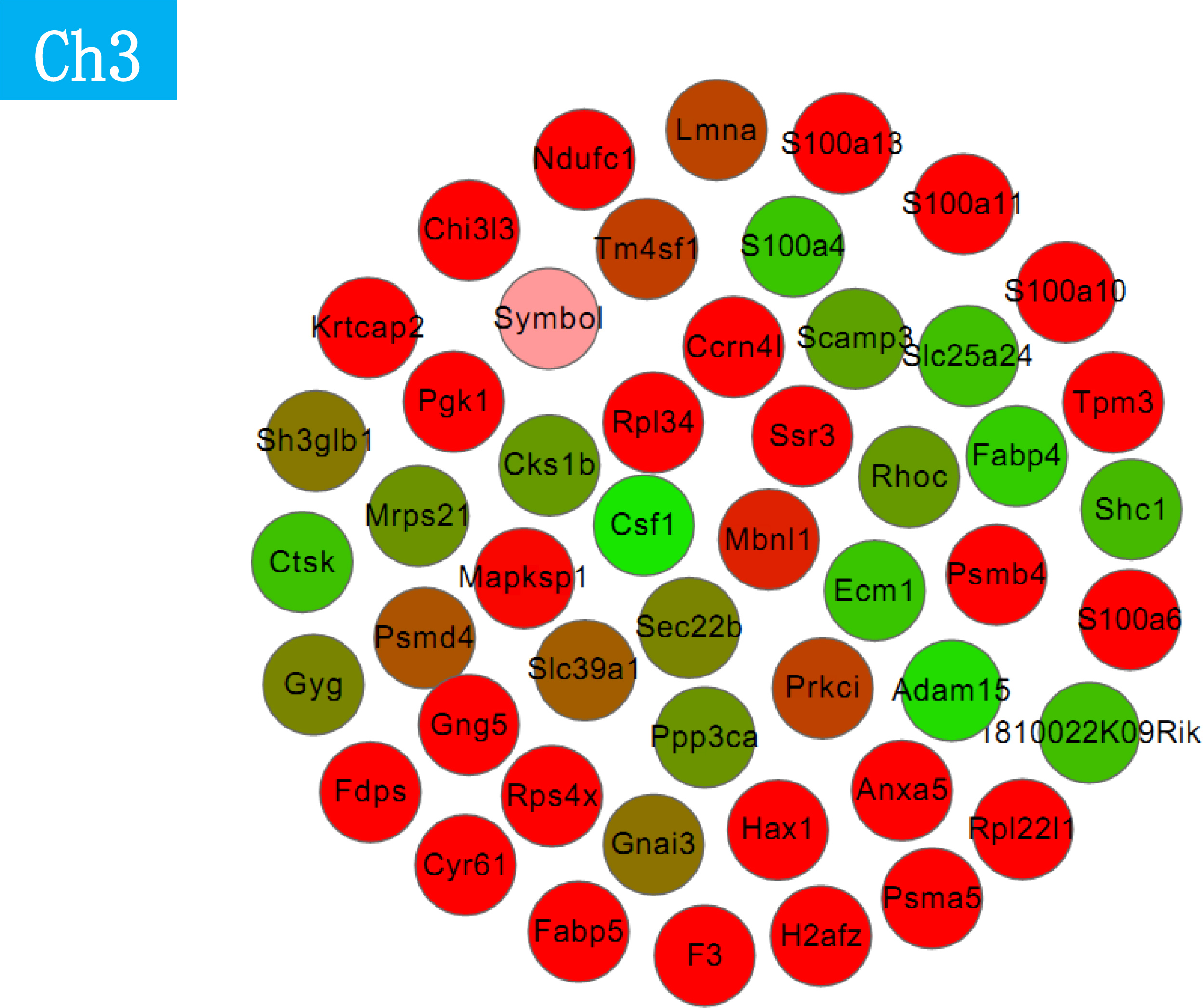

Supplement: Figure S14 — Details of the selected core network genes in mouse alveolar type II cells in chromosome 3. [file jcmm0018-2044-sd16.jpg]

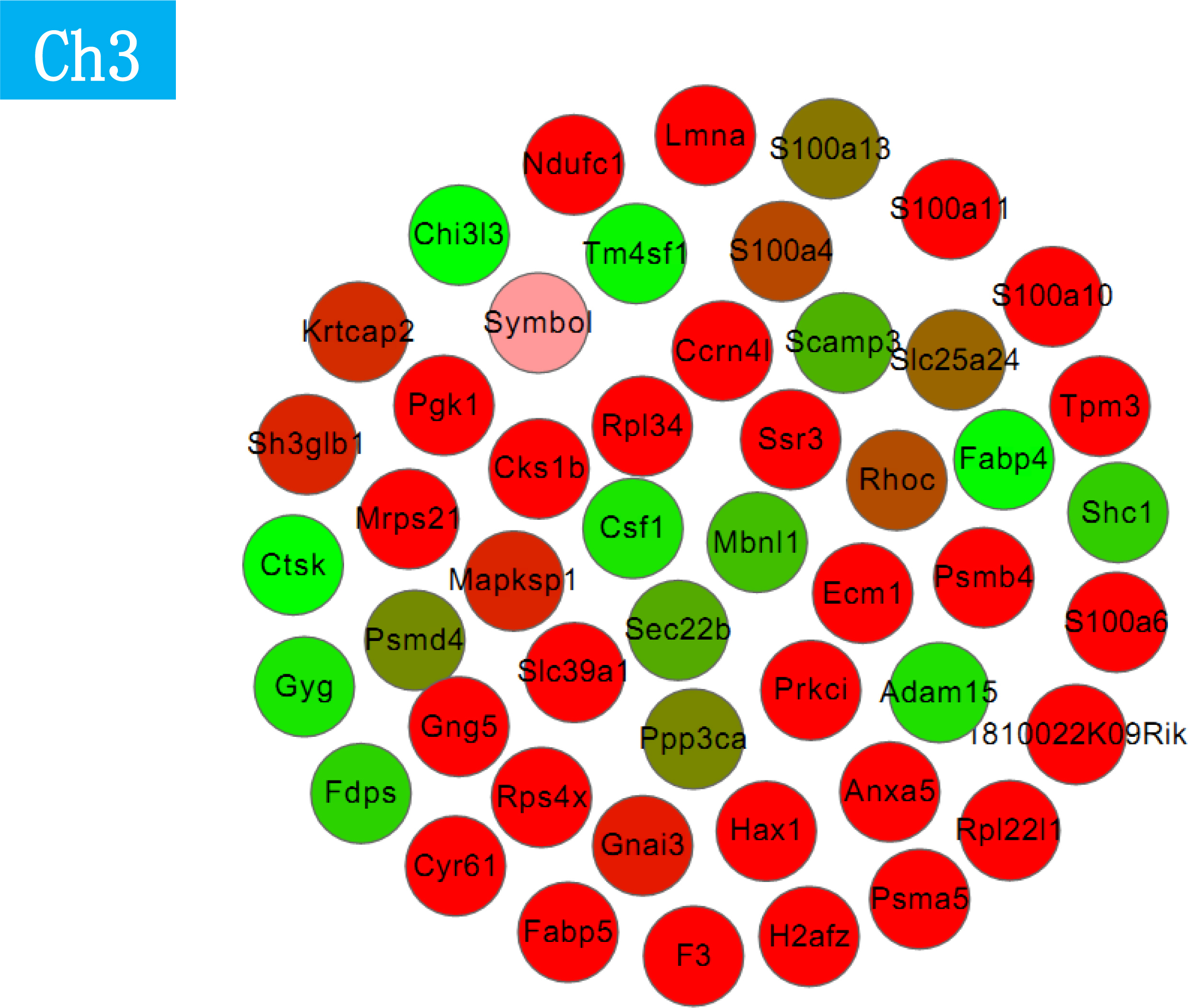

Supplement: Figure S15 — Details of the selected core network genes in mouse airway basal cells in chromosome 3. [file jcmm0018-2044-sd17.jpg]

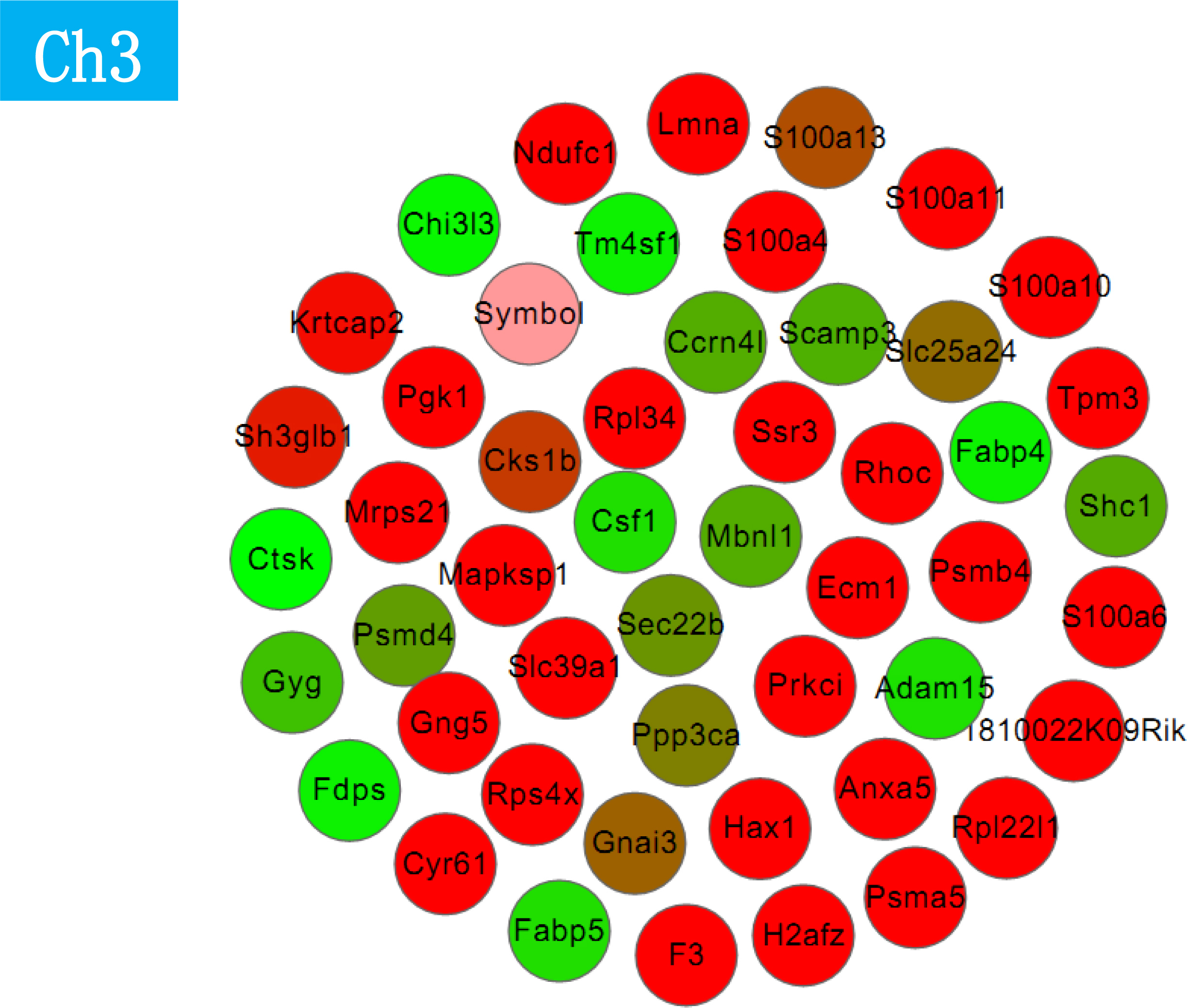

Supplement: Figure S16 — Details of the selected core network genes in mouse proximal airway cells in chromosome 3. [file jcmm0018-2044-sd18.jpg]

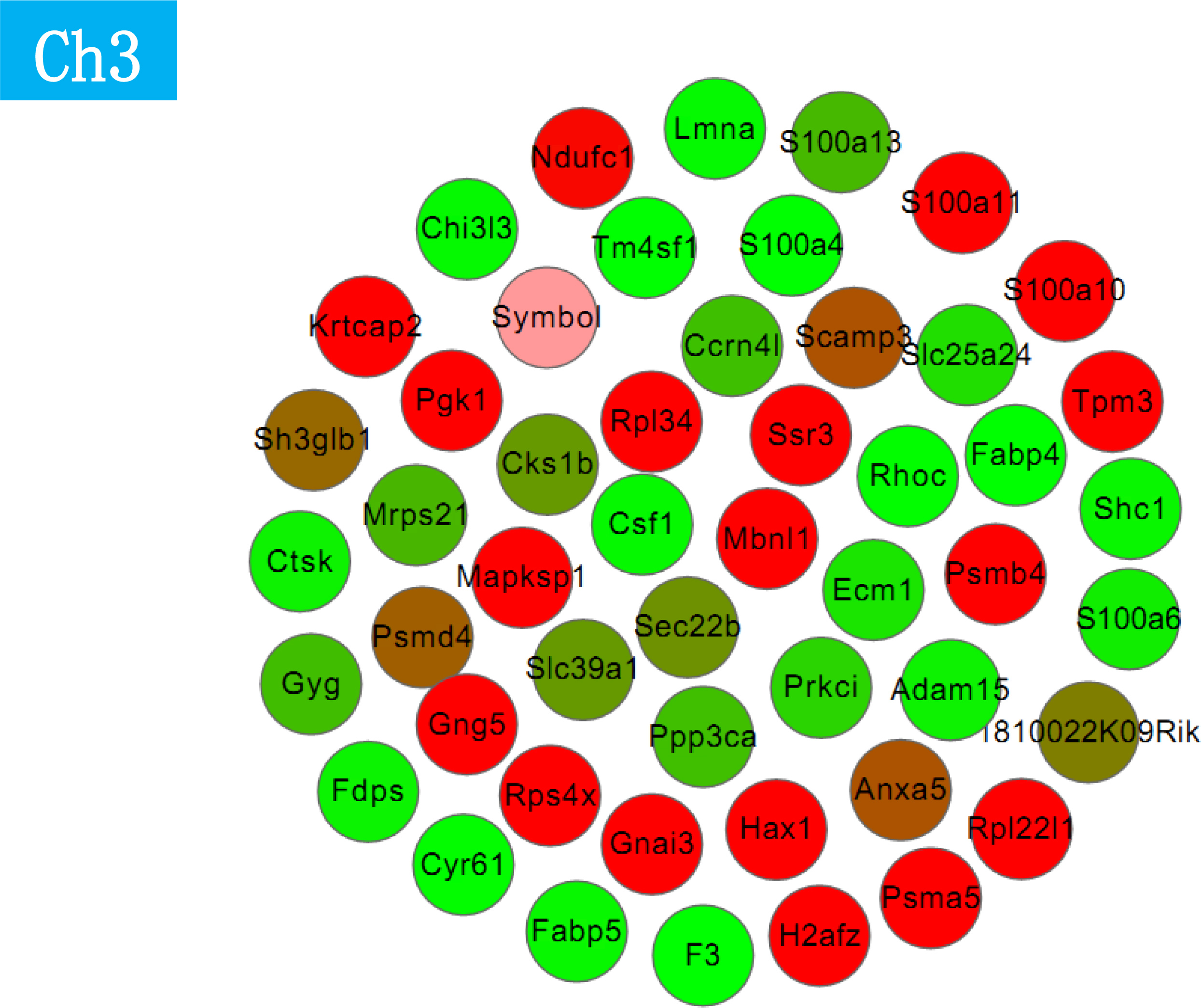

Supplement: Figure S17 — Details of the selected core network genes in mouse CD8+ T cells come from bronchial lymph nodes in chromosome 3. [file jcmm0018-2044-sd19.jpg]

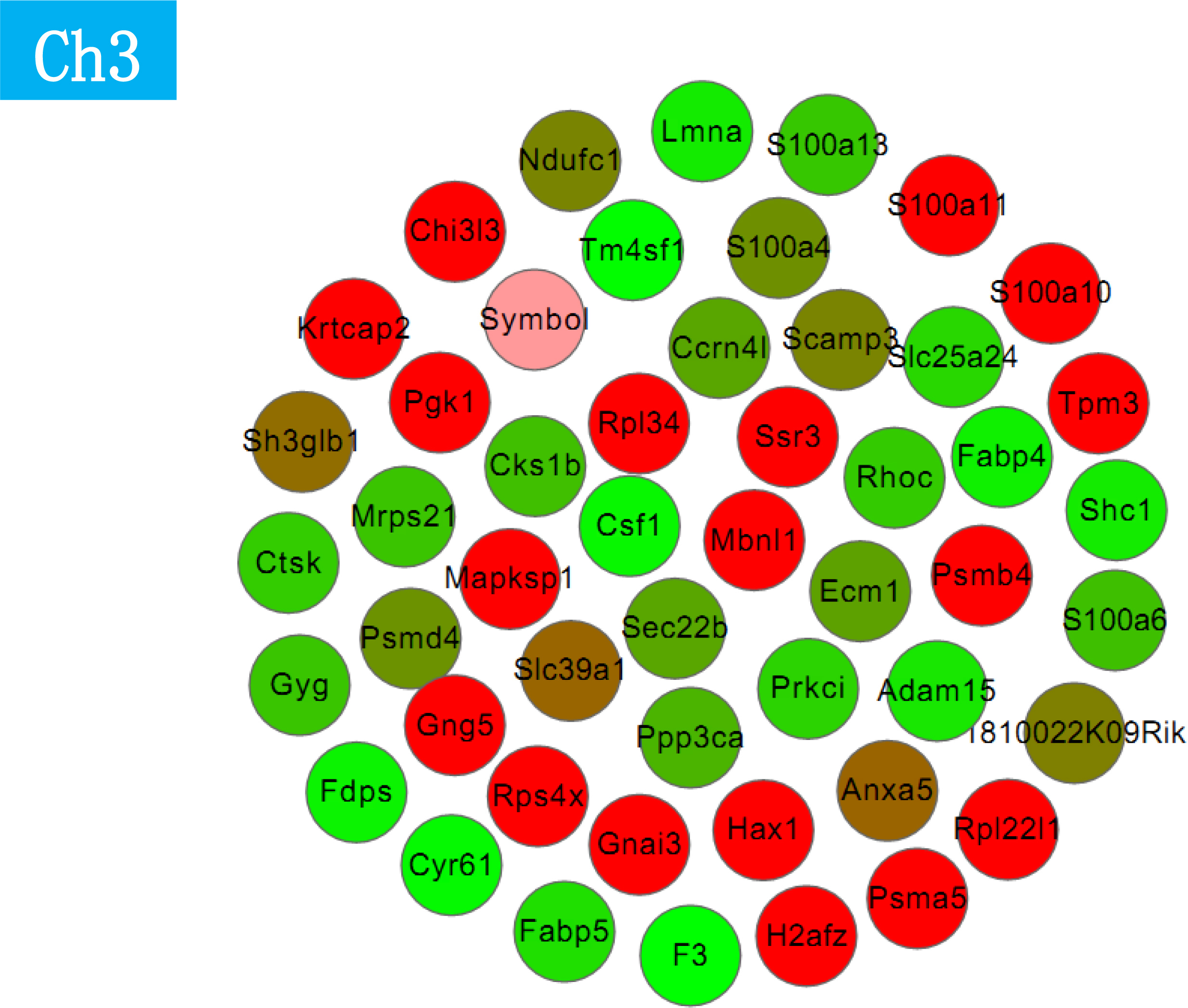

Supplement: Figure S18 — Details of the selected core network genes in mouse CD8+ T cells from lung in chromosome 3. [file jcmm0018-2044-sd20.jpg]
